# Children's Brain Responses to Optic Flow Vary by Pattern Type and Motion Speed

**DOI:** 10.1371/journal.pone.0157911

**Published:** 2016-06-21

**Authors:** Rick O. Gilmore, Amanda L. Thomas, Jeremy Fesi

**Affiliations:** 1 Department of Psychology, The Pennsylvania State University, University Park, PA, United States of America; 2 Department of Psychology, The City College of the City University of New York, New York, NY, United States of America; Durham University, UNITED KINGDOM

## Abstract

Structured patterns of global visual motion called optic flow provide crucial information about an observer's speed and direction of self-motion and about the geometry of the environment. Brain and behavioral responses to optic flow undergo considerable postnatal maturation, but relatively little brain imaging evidence describes the time course of development in motion processing systems in early to middle childhood, a time when psychophysical data suggest that there are changes in sensitivity. To fill this gap, electroencephalographic (EEG) responses were recorded in 4- to 8-year-old children who viewed three time-varying optic flow patterns (translation, rotation, and radial expansion/contraction) at three different speeds (2, 4, and 8 deg/s). Modulations of global motion coherence evoked coherent EEG responses at the first harmonic that differed by flow pattern and responses at the third harmonic and dot update rate that varied by speed. Pattern-related responses clustered over right lateral channels while speed-related responses clustered over midline channels. Both children and adults show widespread responses to modulations of motion coherence at the second harmonic that are not selective for pattern or speed. The results suggest that the developing brain segregates the processing of optic flow pattern from speed and that an adult-like pattern of neural responses to optic flow has begun to emerge by early to middle childhood.

## Introduction

Structured patterns of global visual motion called optic flow provide crucial information about an observer's speed and direction of self-motion and about the shape and trajectory of moving objects [1, 2]. Optic flow patterns change in accordance with the observer's direction and speed of motion and the geometry of the environment. In a rigid environment, optic flow consists of a combination of three components—radial expansion or contraction, rotation, and translation [3]. Forward locomotion produces a radially expanding flow pattern [1, 2]. Head and eye translations yield translational flow while head and eye rotations result in rotational flow. This mapping between optic flow and self-motion provides observers with visually-based proprioceptive information. For example, by locating the central vanishing point in a radial pattern of flow called the focus of expansion, an observer can precisely determine her instantaneous direction of heading [4, 5]. Observers move their eyes, head, and body simultaneously, thus generating complex mixtures of the basic flow types. Nonetheless, adults accurately assess heading direction even under these circumstances [6–9] combining flow with other cues [10–12].

### Neural networks for processing optic flow

Global visual motion patterns caused by self- or object motion activate a network of brain areas in humans and monkeys [1, 13–22]. Depending on stimulus parameters such as speed and pattern type, the areas activated include retinotopic cortex (V1, V2, V3A; [23–26]), V4 [27], the middle temporal (MT/V5; [13, 28]) area and its human analogue (hMT+; [29–35]), the medial superior temporal area (MST; [15, 29, 30, 36]), V6 [37], the lateral occipital cortex (LOC; [13, 15, 18–20, 38]), the ventral intraparietal area (VIP; [39]), the superior temporal sulcus (STS; [24, 26, 37, 38, 40, 41]), and the posterior cingulate cortex [37, 39]. The regions show a degree of functional specialization by motion type (local vs. global, self-motion vs. object motion vs. biological motion, radial vs. rotational vs. translational flow) and sensitivity to non-visual (eye movement, vestibular, auditory) signals, but consensus about how the mature brain's motion sensitive regions combine to serve multiple behavioral tasks remains elusive. Moreover, we lack a complete understanding about how motion processing networks develop.

### Development of optic flow processing

Extensive evidence suggests that primate visual functions, including motion processing, undergo an extended period of postnatal development [42–47]. Differences in the spatial patterns of motion and speeds used by different research groups complicate the task of summarizing the pattern of findings. Nevertheless, sensitivity to optic flow emerges early [48], develops relatively slowly [22, 44], and the rate of development varies by motion type and speed.

Specifically, infants initially show higher sensitivity to *translational* motion and faster motion speeds, but gradually acquire greater sensitivity to *radial* and *rotational* motion at slower speeds. For example, macaques show prolonged development of behavioral sensitivity to *translational* motion [44], with adult-like responses emerging only in adolescence. The youngest monkeys show peak sensitivity to fast (20–30 deg/s) speeds while adults show peak sensitivity at slow to moderate (3–6 deg/s) speeds [44]. Sensitivity to translational motion at slow to moderate (1–4 deg/s) speeds is also reduced in (5–6 year-old) human children relative to adults [49, 50]. Similarly, human infants' sensitivity to detect *radial* optic flow displays develops slowly over the first year of life [51], with higher sensitivity to expansion patterns relative to contraction. The ability to discriminate radial from translational flow emerges between two and three months at slow to moderate (2.7–5.3 deg/s) speeds [52]. But, two- to three-month-olds appear insensitive to rotation except at fast speeds (>10 deg/s), and there is no change in sensitivity across this time period [53]. Radial optic flow sensitivity develops throughout childhood into adolescence, showing immaturity relative to adults even at 16 years of age [54]; both children and adults show greater sensitivity to 5.5 deg/s radial motion relative to 1.6 deg/s motion. The development of motion processing continues throughout childhood [42, 47, 49–50, 54–59]. For example, behavioral sensitivity to the direction of translational motion continues to improve throughout adolescence [57–59]. Radial, rotational, and translational motion patterns by definition differ in terms of their distributions of motion direction. Thus, the detection and integration of motion direction differences must contribute to the ability to discriminate different types of optic flow patterns from one another.

These results imply that the immature primate perceptual system responds most strongly to motion speeds greater than 5 deg/s, and that the development of sensitivity to different patterns of motion occurs at different rates. Slow maturation of motion processing specific neural networks may underlie these patterns of development [44] and these may be driven in part by changing statistics of visual input [60, 61] that are initially biased toward fast linear flows. Whether development in motion processing is best characterized by changes in speed sensitivity or a combination of spatial and temporal sensitivity remains a point of active research [44, 47, 62–64]. Equally important, in our view, is the extent to which changes in motion perception result from development in brain systems linked to motion processing.

#### Development of brain responses to flow

**Fig 1:**
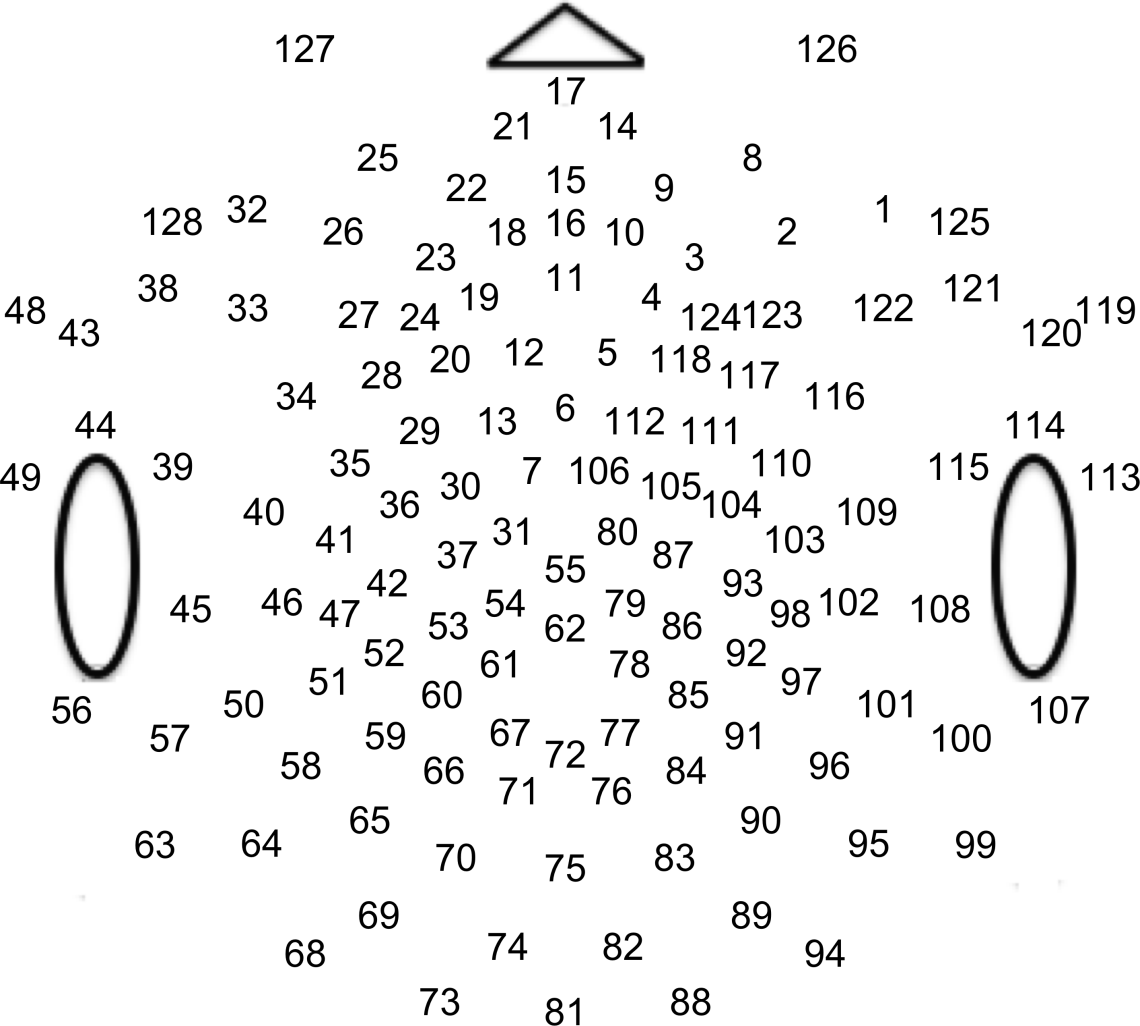
Channel map of EGI HydroCel Geodesic Sensor Net. The numbers depict the channel indices used for subsequent plots.

Data about brain responses to optic flow across development largely support the behavioral evidence. Four- to six-month-old human infants show stronger steady-state visual evoked potential (SSVEP) responses to (5.5 deg/s) direction-reversing translational motion than to either radial or rotational motion [63] while adults show the reverse pattern. Infant brain responses cluster over midline channels (Oz, comparable to channel 75, see Fig 1), while adults show larger responses over lateral channels (PO7, O1, O2, and PO8; comparable to channels 65, 70, 83, and 90 in Fig 1) [63]. Further, infants' evoked responses over midline channel Oz to coherence modulating rotational flow peak at large displacements and speeds (20–30 deg/s) whereas adult responses peak at smaller displacements and speeds (6–8 deg/s) [63]. Similarly, event-related potentials (ERPs) to rotational motion from electrodes positioned over lateral motion-sensitive areas grow in magnitude from 3 to 5 months [65]. Three- to four-month-old infants show cortical responses to (6.8 deg/s) radial flow, but stronger responses to contraction than expansion [66]. Brain responses to radial motion mature across the first year [67]. Brain response data from children are limited, but show evidence of differential responses by motion pattern. Radial patterns evoke stronger evoked responses than translational motion in 10–12 year olds [68], but Kubova et al. [69] found large variation in the detectability and timing of EEG responses to the onset of translational and radial motion in a sample of 7–12 year old children. To our knowledge, there are no existing published data reporting on evoked brain responses to different patterns and speeds of optic flow in preschool or early school-age children. So, many questions remain about how children's brain responses to different flow patterns and speeds develop between infancy adulthood.

However, a recent study with adults suggests the target of this developmental trajectory. Fesi and colleagues explored the effects of optic flow pattern and speed (2–16 deg/s) on SSVEP responses in adults [17]. Radial flow evoked the strongest responses, and the location of activity varied by speed. Slow radial motion elicited activation in lateral occipital channels and fast radial motion activated dorsomedial channels. Rotation and translation patterns showed medial occipital channel activation for slow speeds and a similar pattern of dorsomedial activation for fast speeds.

The current study investigates the patterns of evoked brain activity to motion in preschool and early school age children (4–8 years of age). The aim is to determine whether children show evoked brain responses to motion that differ by pattern type and by speed. Do young children show larger amplitude responses to radial motion like adults [17] and adolescents [68], or stronger responses to translational motion like infants [62]? Do children's evoked brain responses differ by speed like adults [17]? What is the spatial distribution of pattern and speed-sensitive activation in the brains of young children? Is it clustered over the central midline as some [62, 64] but not all [22], prior research with infants would suggest, or is it distributed laterally as some [17] but not all [22] prior research with adults would suggest?

## Methods and Materials

### Participants

Thirty-three children, (15 female) between four and eight years of age participated in the study. The mean age was 75.48 months (*SD* = 19.18). The sample consisted of children drawn from a database of families in Centre County, Pennsylvania. Children were excluded if they were born prematurely, had a history of serious visual or medical problems, epilepsy, or seizures. All children tested had normal pattern vision as evaluated with Teller Acuity Cards, a measure of visual function designed for non-verbal participants. We obtained written consent to participate from parents or guardians on behalf of the children and written assent from the children themselves under procedures approved by the Institutional Review Board of The Pennsylvania State University (#37946). The research was conducted according to the principles expressed in the Declaration of Helsinki.

### Stimuli

**Fig 2:**
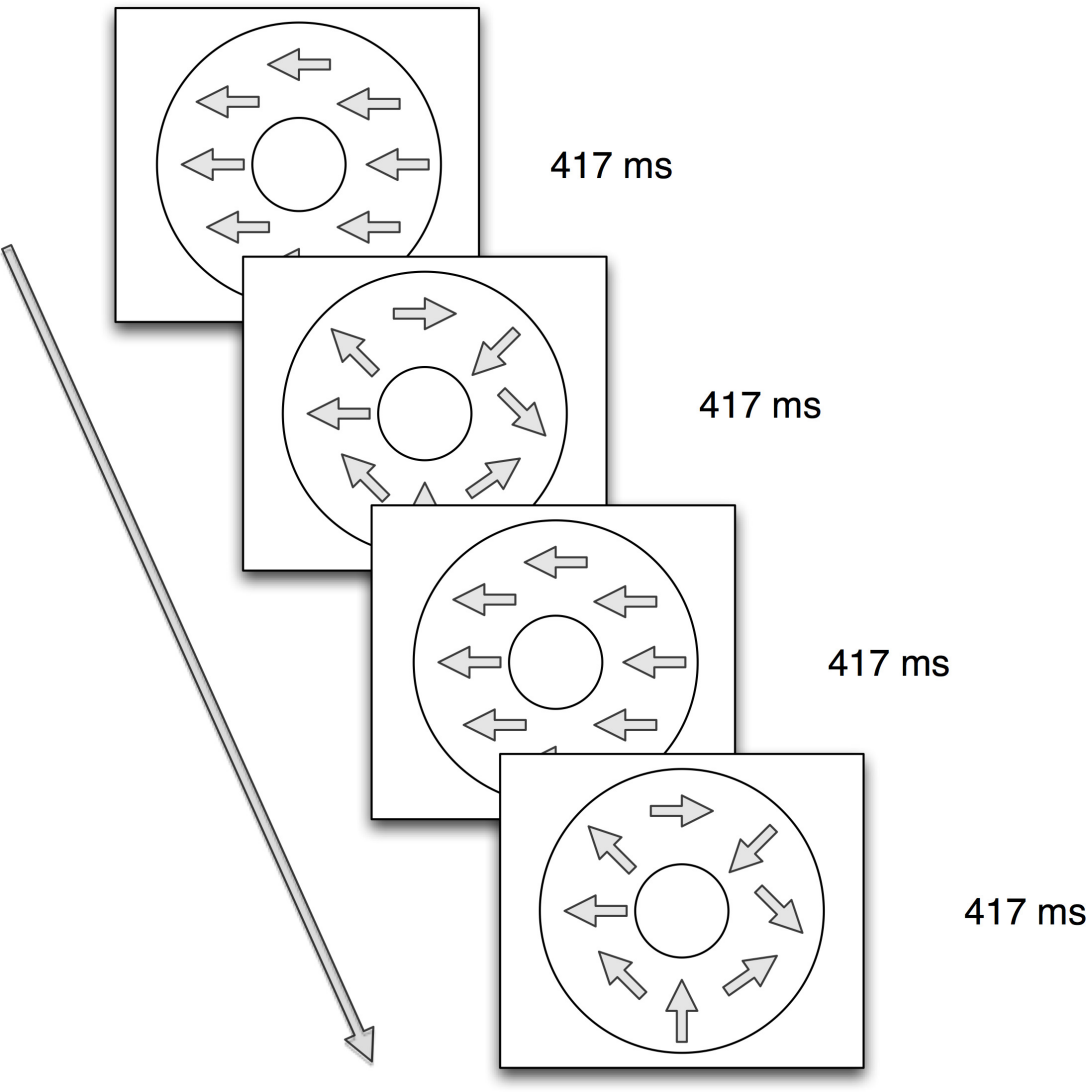
Schematic of Optic Flow Display. Displays alternated between a (100%) coherent phase with radial, rotational, or translational global motion and an incoherent (0% coherent) phase at a fundamental frequency (1F1) of 1.2 Hz. In the actual displays, white dots were presented on a black background. For movies of sample displays and links to the full and extended dataset, see http://doi.org/10.17910/B7QG6W.

Participants viewed limited lifetime random-dot kinematograms generated by a Macintosh G4 computer using PowerDiva Video software (version 3.4, Smith-Kettlewell Eye Research Institute) connected to a monochrome Mitsubishi Std Diamondtron 2060u monitor with an 800 x 600 pixel resolution and a refresh rate of 72 Hz. All patterns were displayed in an annular region 24 deg in outer and 4.8 deg inner diameter at the 60 cm viewing distance. The display consisted of white (79.4 cd/m^2^) dots on a black (6 cd/m^2^) background. Dots were 7 arc min (.12 deg) in diameter and plotted at a density of 7.35 dot/deg^2^. The displays alternated between globally coherent (100% coherent) and globally incoherent (0% coherent) motion every 417 ms. A complete on/off cycle lasted 833 ms, for a fundamental frequency (1F1) of 1.2 Hz (See Fig 1). The frame rate for updating dot positions was 24 Hz (1F2); dot positions were updated every 3 screen refresh cycles. During incoherent motion, the range of possible dot directions on each update cycle was 360°. During coherent motion dot directions on each update were specified by the type of global motion pattern condition. Three types of global motion patterns were displayed—left (counterclockwise) and right (clockwise) rotation, left and right translation, and radial expansion and contraction. In order to reduce response adaptation, the direction of motion reversed every other cycle (at 0.6 Hz). Each pattern was shown at three speeds: 2, 4, and 8 deg/s, forming a total of nine conditions. Dot speeds in both the incoherent and coherent phases within a motion speed condition were identical. Each trial consisted of 10 cycles of coherent/incoherent motion. Four to 10 trials per condition were collected contingent on participant comfort and compliance in a single laboratory testing session. The maximum dot lifetime was 100 dot updates (4.17s); 1% of the dots were repositioned on each update on a fixed schedule. The relatively long dot maximum lifetime was used to reduce low frequency luminance artifacts that can result from more frequent dot repositioning. Fig 2 shows a schematic of the display.

### Procedures

Upon arrival at the laboratory, study and visit procedures were described and informed consent/assent was obtained. After net fitting and placement, participants were escorted to the testing room and seated on an adjustable chair in front of the computer monitor. Electrode impedances were checked, and once impedances met testing criteria (at 50kΩ or below), lights were dimmed and the session began. A research assistant remained in the testing room to monitor participants' fixation and to call for breaks, as needed. The entire testing session took about 45 min.

#### EEG Collection

A 128-channel *HydroCel Geodesic Sensor Net* (Electrical Geodesics, Inc.) was used in conjunction with NetStation 4.1 software to record SSVEP responses to the stimuli. EEG was sampled rate of 432.43 Hz, referenced to Cz, and the signal was low-pass filtered at 50 Hz [70] prior to analysis. Amplitude modulations that exceeded 60μV were rejected as artifact. Trials that had 15% of coherent/incoherent cycles rejected by these criteria were excluded from analysis. Children who produced fewer than three trials per condition were also excluded. A total of four children were excluded from analysis; two were excluded due to too few trials and two others were excluded due to equipment malfunction. In total, 29 children were included in the analysis (mean age = 75.48 months; *SD* = 19.18).

### Analysis

PowerDiva Host 3.4 software was used offline to analyze the cortical activity related to the display of the stimuli. The software extracted frequency domain components at low order integer harmonics of the fundamental frequency (1F1 = 1.2 Hz) of the motion coherence modulation and the dot update rate (1F2 = 24 Hz) using a discrete Fourier transform algorithm. Data analysis focused on the phase-locked amplitude of responses at 1F1, 2F1, 3F1 and 1F2 [17, 62, 64, 70]. Phase-locked coherent averages of response amplitudes were analyzed and visualized using R 3.2 [71], RStudio 0.99.446 (http://www.rstudio.com), and the packages *ggplot2* [72] and *dplyr* [73]. To reduce the likelihood of false positives, a conservative mass univariate approach [74] was utilized in which we analyzed speed and pattern effects separately for each individual channel while choosing a conservative criterion for determining statistical significance. To evaluate the overall responsiveness of EEG channels to motion coherence modulations, we calculated for each channel the T2Circ statistic [75], a version of Hotelling's T2 statistic adapted for frequency domain data, across participants, pattern types, and speeds. It is analogous to conducting a *t* test with the null hypothesis that EEG amplitudes are equal to zero. Since the T2Circ statistic could cause us to overlook channels that responded to the pattern and speed conditions with distinctive amplitude and phase profiles, we also computed a mixed-effects MANOVA (using R's *manova* command). Here, the real (cosine) and imaginary (sine) components of the Fourier decomposition of the EEG signal served as outcome variables. This revealed those EEG responses that were time/phase-locked to the stimulation modulations. We chose this approach in order to evaluate jointly amplitude and phase differences in the evoked responses while avoiding distributional complications with analyses based on amplitudes and phases. Specifically, amplitudes have a fixed floor at zero voltage, and phases are circularly distributed. Thus, both of these polar space quantities violate distributional assumptions that underlie standard linear statistical models. The real and imaginary components of the EEG are, under the null hypothesis, distributed symmetrically around zero, consistent with the distributional assumptions that underlie the general linear model.

Consistent with standard mixed-effects modeling procedures in the behavioral sciences, participants' mean response levels were included in the model as random intercept effects to attempt to control for individual differences in EEG response magnitudes. We used the default Pillai trace test that approximates an *F* distribution as the statistic of interest. To interpret the results, we chose a statistical criterion of *alpha* = .0005. This compares to a Bonferroni adjustment with *n* = 100 tests, assuming a baseline criterion of *p* < .05. We report partial *η*^2^ values to indicate effect sizes for the MANOVA results. For illustrative purposes we plotted the channel-wise results over a range of strict and liberal levels of statistical significance to illustrate how the effects varied by choices of criterion. Additional figures, full datasets, and links to analysis code may be found at http://doi.org/10.17910/B7QG6W.

## Results and Discussion

Analyses proceeded in three steps. We first estimated overall effects of age, pattern, speed, and channel in an omnibus analysis. Then in separate analyses we evaluated the phase-locked EEG responses at three harmonics of the frequency of the on/off coherence modulation (1F1 or 1.2 Hz; 2F1 or 2.4 Hz; and 3F1 or 3.6 Hz), and the fundamental frequency of the dot update rate (1F2 or 24 Hz). Finally, we compare the results from children to those of a separate, previously collected sample of adults tested on the same displays.

### Omnibus results

As the study was designed primarily to examine the within-subjects effects of pattern, speed, and channel we had no prior hypotheses about age-related trends. Nevertheless, to address the possibility that there were significant age-related effects, we carried out a multivariate analysis of variance (MANOVA) with the real and imaginary components from the 1F1 harmonic of the EEG as the response measure and pattern type (radial, rotational, linear), speed (2, 4, and 8 deg/s), channel (1–128), and participant age in weeks as predictors. This analysis showed no effect of participant age, *F*(2, 32255) = 0.0564, *n*.*s*., but significant effects of channel, *F*(254, 32255) = 19.55, *p*<2.2e-16 qualified by significant channel by pattern, *F*(508, 32255) = 2.49, *p*<2.2e-16, and channel by speed, F(508, 32255) = 1.28, *p*<2.23e-5 interactions. A parallel analysis on the dot update rate (1F2) harmonic showed effects for channel, *p*<2e-16 and the speed by channel interaction, *p*<2e-16. Therefore, in the subsequent analyses, we dropped age as a predictor to focus on analyzing the speed and pattern effects by channel for the sample as a whole.

### Motion coherence results (1F1)

**Fig 3:**
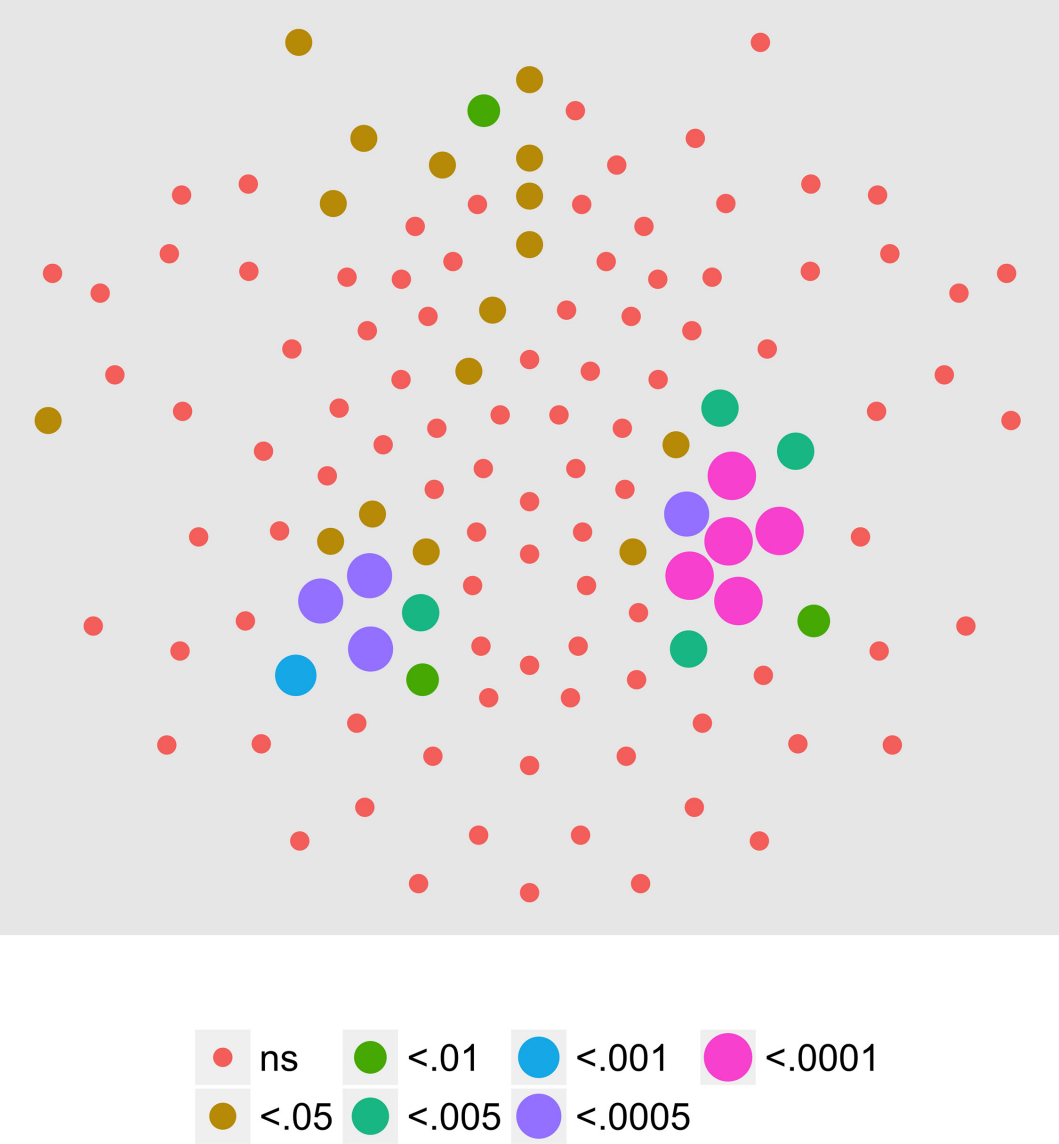
T2Circ Values by Pattern and Speed -- Children -- 1F1. Plots of individual channel-level effects from a T2Circ statistic computed on the real and imaginary EEG components at the first harmonic (1F1) of the coherence modulation frequency (1.2 Hz) for each of the three pattern (rows) and speed (columns) conditions. See Fig 2 for details about channel locations and labels.

We next examined the extent to which on/off motion coherence modulations resulted in phase-locked EEG activity to any of the pattern and speed manipulations. Fig 3 plots the results of a channel-wise analysis of the T2Circ statistic. It shows that there are 9 channels that meet the *p* < .0005 criterion distributed in clusters located over right central and left central/posterior channels.

**Fig 4:**
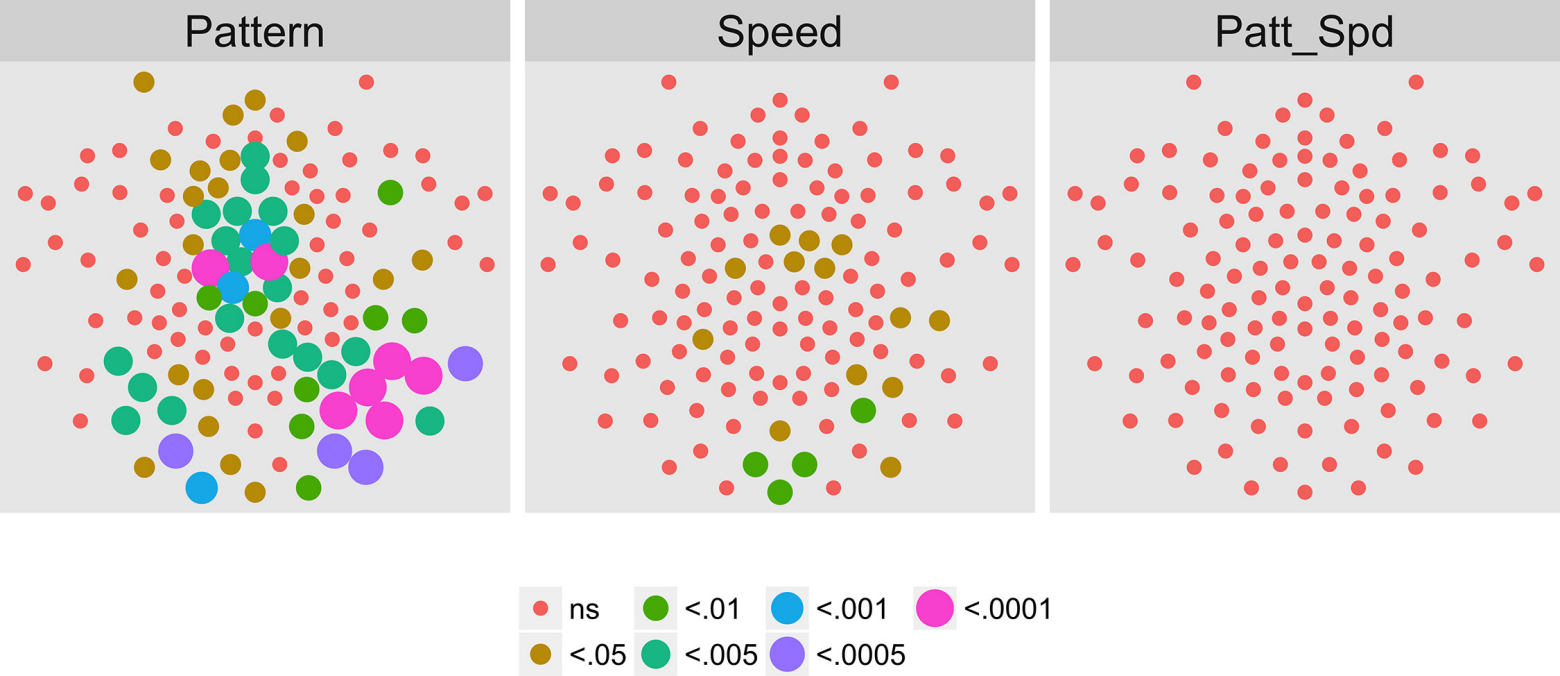
Channel-wise MANOVA on 1F1. Plots of individual channel-level effects from a MANOVA on the real and imaginary EEG components at the first harmonic (1F1) of the coherence modulation frequency (1.2 Hz). At the pre-selected threshold of *p* < .0005, responses show an effect of pattern over a cluster of right lateral and mid-central channel locations. Effect sizes were small to medium, with partial *η*^2^ values ranging from 0.046–0.069. See Fig 1 for details about channel locations and labels.

We then asked which channels responded differently—in magnitude or phase—to the patterns and speeds. To answer this question, we conducted a mixed-effects MANOVA. The real and imaginary components from the 1F1 harmonic served as the response measures while pattern and speed served as predictors. We fit the identical model to each of the 128 separate EEG channels. Fig 4 depicts the results of this channel-wise analysis. It shows the channels that met varying uncorrected significance levels for the pattern (left panel) and speed (center panel) effects and for the pattern by speed interaction (right panel). Statistical results for each channel are reported in the supplemental materials (S1 Table). As the figure indicates, all 11 channels that met criterion did so for the pattern contrast. There were no statistically significant effects for speed or for the speed by pattern interaction. The figure also indicates that the channels showing statistically significant variation by optic flow pattern clustered over right lateral areas. Two were located in the mid-dorsal area, and there was a single left lateral channel. None of the channels that met the *p* < .0005 criterion in the T2Circ analysis (Fig 3) were among those showing a significant multivariate effect for pattern. This implies the existence of circuits responsive to motion coherence modulations in general that differ from those responsive to specific types of motion patterns or speeds.

**Fig 5:**
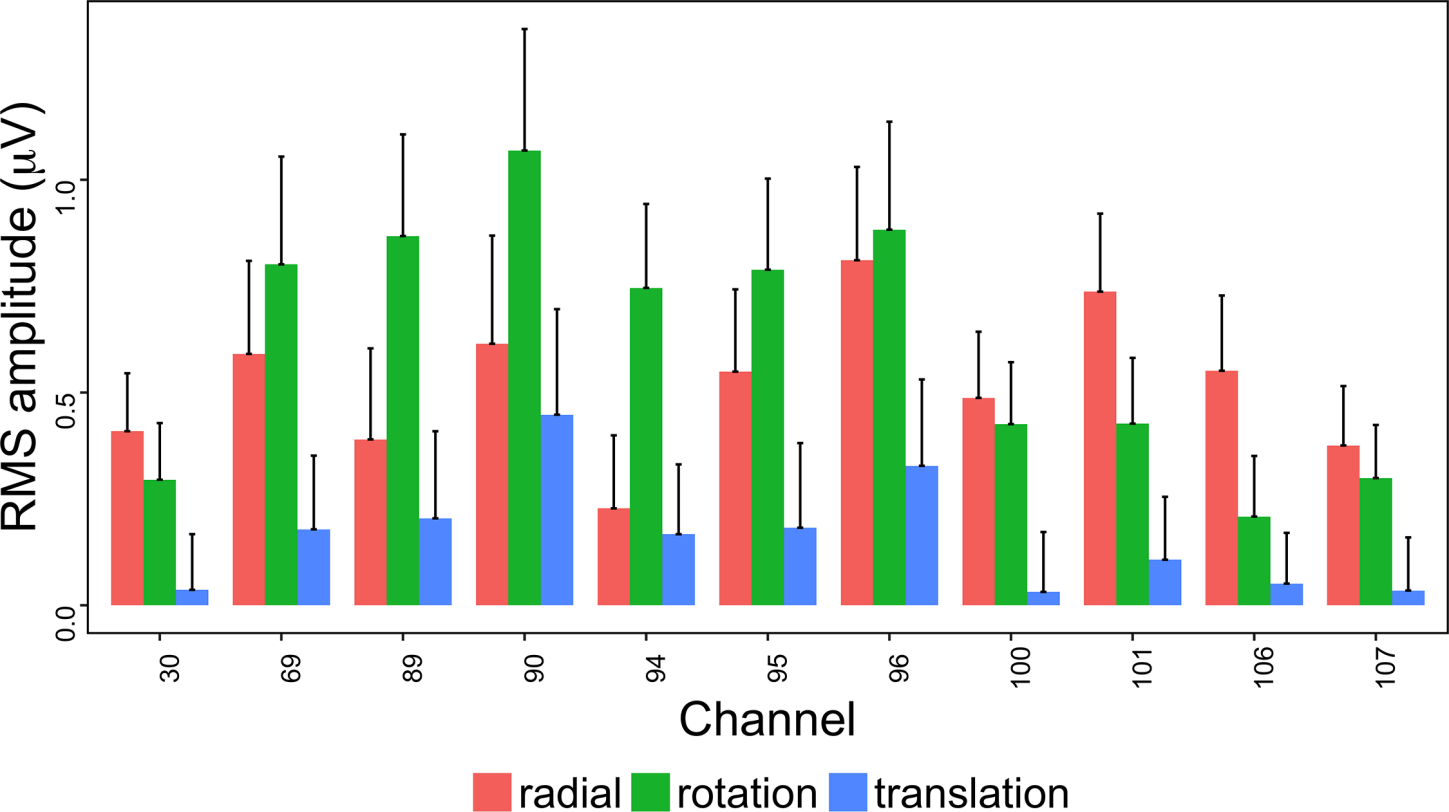
Channels With Significant Pattern-Related Activity at 1F1. Plot of all channel-level pattern effects exceeding the *p* < .0005 threshold. The bars indicate the across-participants average (+ 1 SEM) of the root mean square (RMS) amplitude of the real and imaginary signal components at 1F1 (1.2 Hz) calculated for each participant. In most channels, rotation and radial flow patterns elicit larger responses than translational flow.

To visualize the nature of the pattern effect in these channels of interest, we calculated for each participant and condition the root mean square (RMS) amplitude. Fig 5 shows the across-participants mean (+1 SEM) amplitude for the 11 channels of interest. The figure shows that that radial and rotational flow patterns evoke the largest amplitude responses in most channels and that channels vary in the extent to which pattern type evokes the strongest activity.

**Fig 6:**
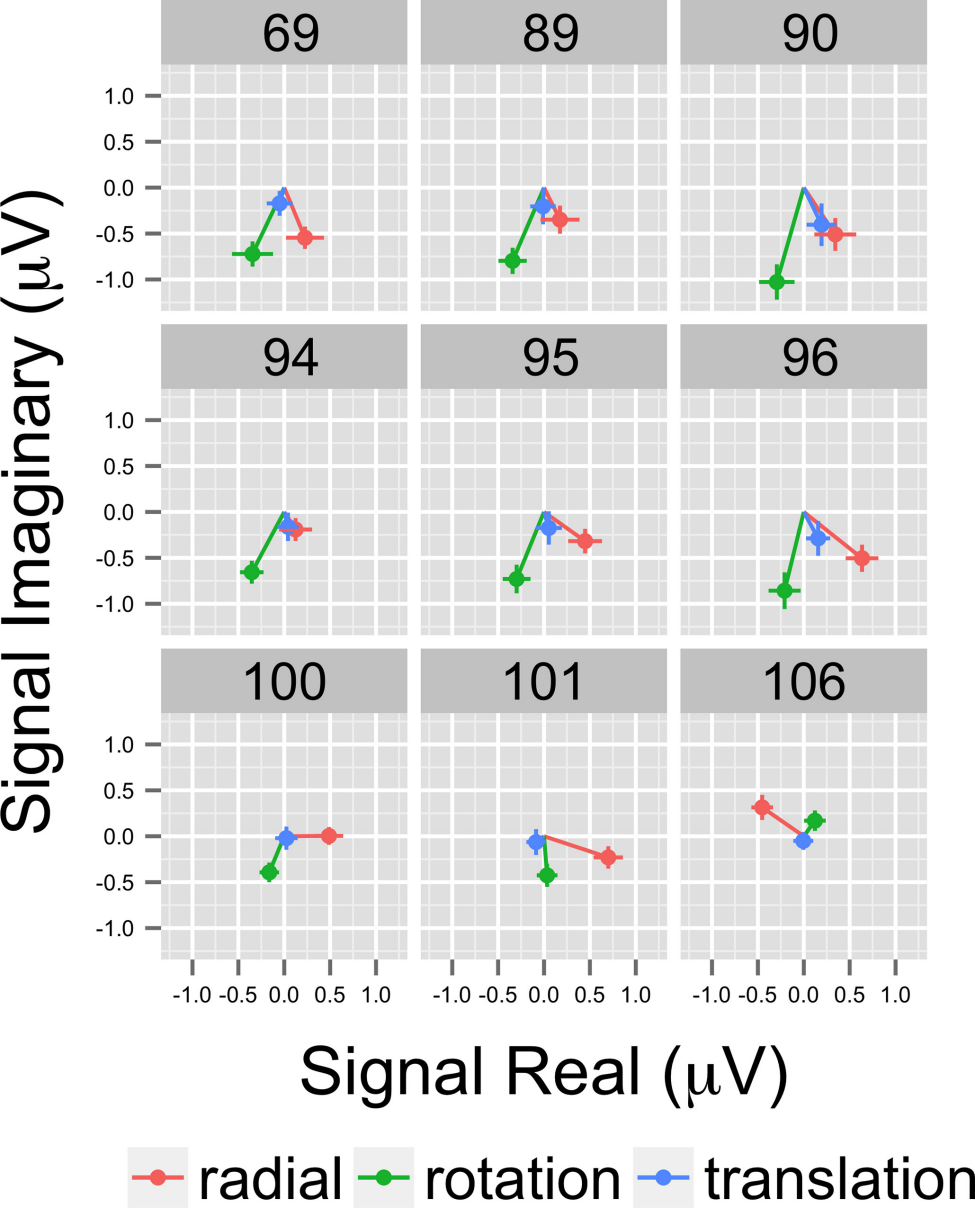
Complex Domain EEG Responses at 1F1 from Illustrative Channels. Vector plots from the nine highest amplitude channels showing the average (+ 1 SEM) real and imaginary EEG signal components. Note that responses differ both in amplitude and phase angle.

Both phase and amplitude differences can contribute to statistically significant effects in the frequency domain. That is one rationale for using MANOVA with the real and imaginary (complex domain) responses for analysis. To visualize them, we selected the nine highest amplitude channels and calculated for each participant the average real and imaginary EEG signal components. This results in a vector for each participant representing the average amplitude and phase of that individual's response by condition. Fig 6 shows a plot of nine channels with the highest overall amplitudes in a form that depicts the vector average response across participants for each pattern condition. In this figure, the distance from the origin represents signal amplitude, akin to Fig 5, and the angular position around the circle represents the relative phase. The figure also shows standard errors of the mean (SEM) for both the real and imaginary components. The figure shows that the average EEG response to motion patterns that modulate in global coherence over time varies in both amplitude and phase depending on the optic flow pattern type (radial, rotational, and translational). The figure shows that responses to translation cluster near the origin in 7 out of 9 channels (channels 90 and 96 are the exception), but activity to radial and rotational flow do not. Note the high degree of consistency between the amplitude-only (Fig 5) and amplitude plus phase (Fig 6) depictions of the data.

#### Motion Coherence Results (2F1)

**Fig 7:**
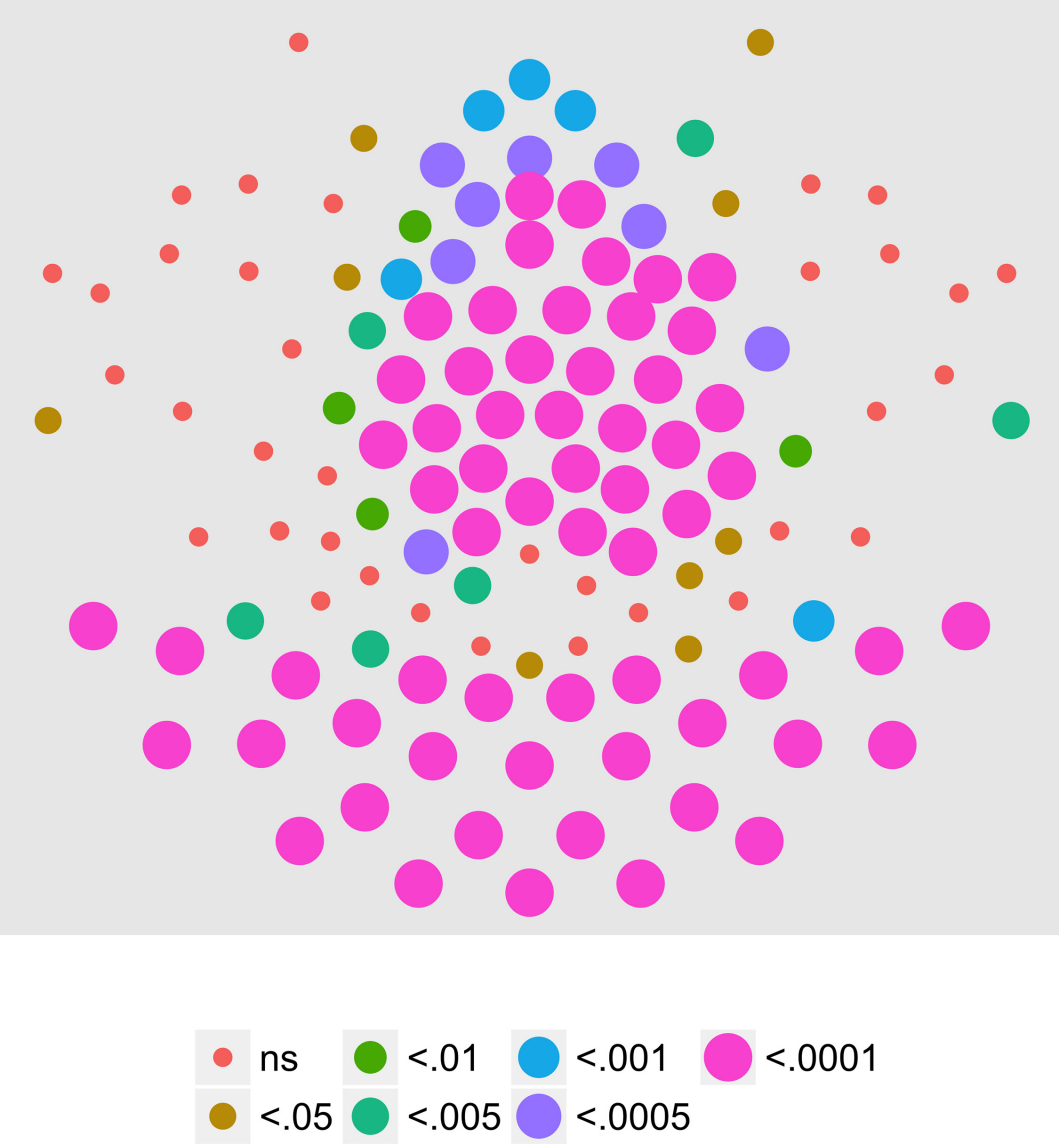
T2Circ Values by Pattern and Speed -- Children -- 2F1. Plots of individual channel-level effects from a T2Circ statistic computed on the real and imaginary EEG components at the second harmonic (2F1) of the coherence modulation frequency (2.4 Hz) for each of the three pattern (rows) and speed (columns) conditions.

We analyzed the second harmonic of the response (2F1) in a fashion identical to that described for the first harmonic. According to a linear systems view, responses at this and higher order *even* harmonics should reflect brain systems sensitive to *changes*, both offset and onset, in motion coherence. Fig 7 shows the results of an analysis of the T2Circ statistic. The figure shows that 69 channels met criterion. These are organized in a large and widespread cluster of bilateral posterior and central frontal channels all of which were activated by the onset and offset of coherent motion at 2F1.

**Fig 8:**
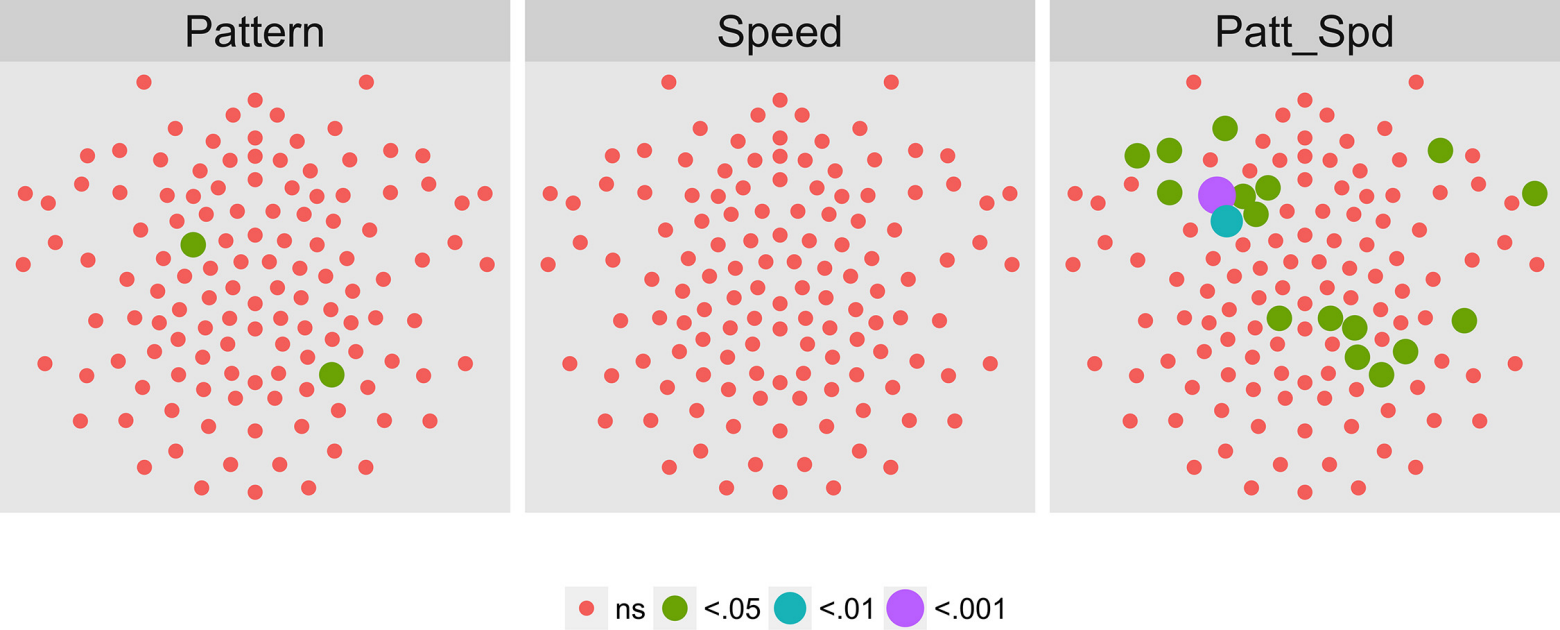
Channel-wise MANOVA on 2F1. Plots of individual channel-level effects from a MANOVA on the real and imaginary EEG components for each condition at the second harmonic (2F1) of the coherence modulation frequency (1.2 Hz). No channels met the pre-selected threshold of *p* < .0005 in any condition.

Fig 8 shows the results of a MANOVA on the 2F1 data. In contrast to the T2Circ analysis, here no channels in any condition met the *p* < .0005 statistical threshold. The fact that we found large numbers of active channels using T2Circ but not the multivariate test illustrates that the two approaches measure different, but complementary, aspects of the brain's response to coherent motion modulations. T2Circ measures a channel's response across patterns and speeds while the multivariate analysis measures selectivity among particular flow patterns or speeds.

#### Motion Coherence Results (3F1)

**Fig 9:**
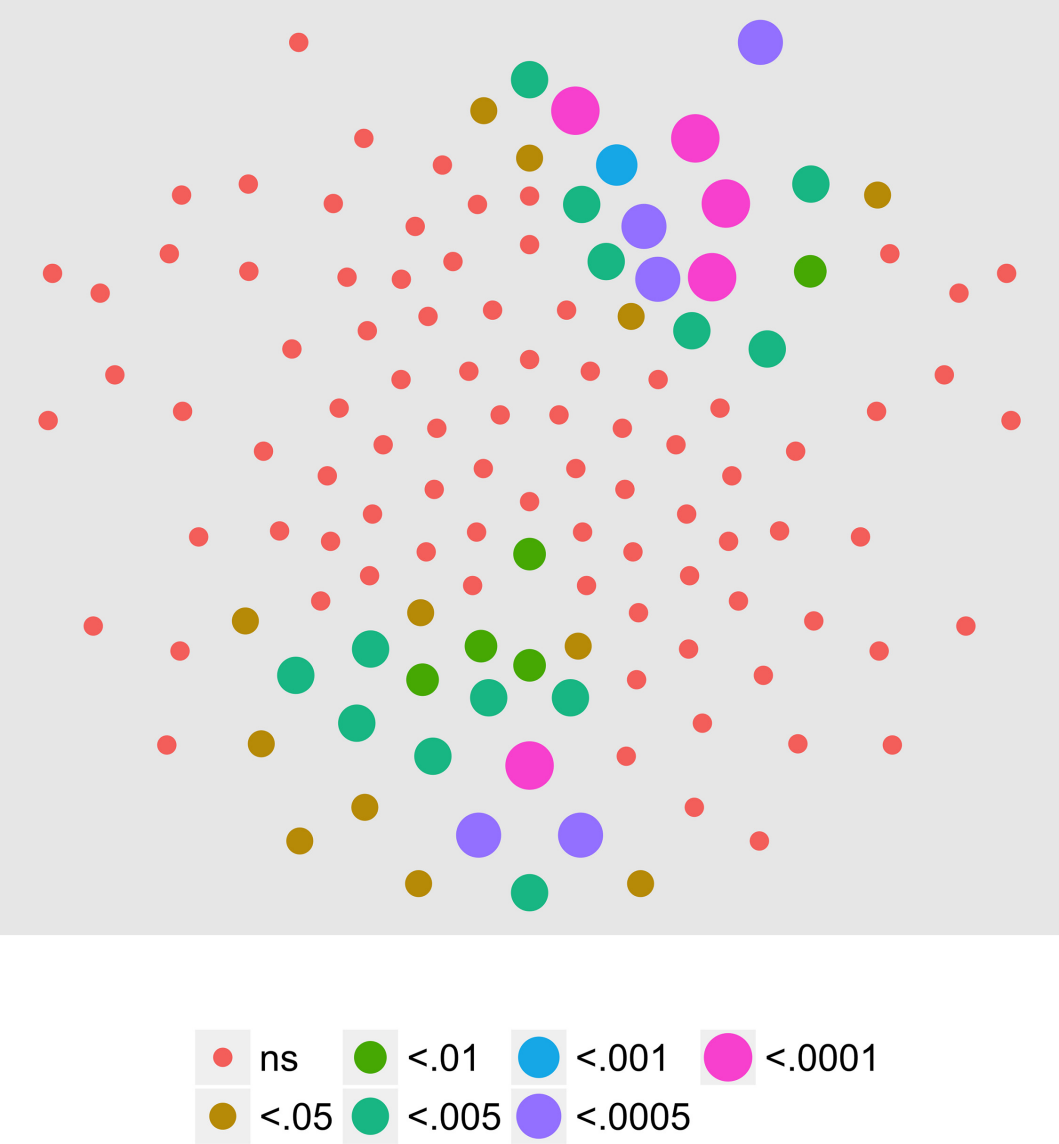
T2Circ Values by Pattern and Speed -- Children -- 3F1. Plots of individual channel-level effects from a T2Circ statistic computed on the real and imaginary EEG components at the third harmonic (3F1) of the coherence modulation frequency (3.6 Hz) for each of the three pattern (rows) and speed (columns) conditions.

Fig 9 shows the results of an analysis of the T2Circ values by channel, speed, and pattern condition for the third harmonic (3F1, 3.6 Hz) of the motion coherence modulation. Ten channels clustered over the posterior and right anterior midlines met criterion.

**Fig 10:**
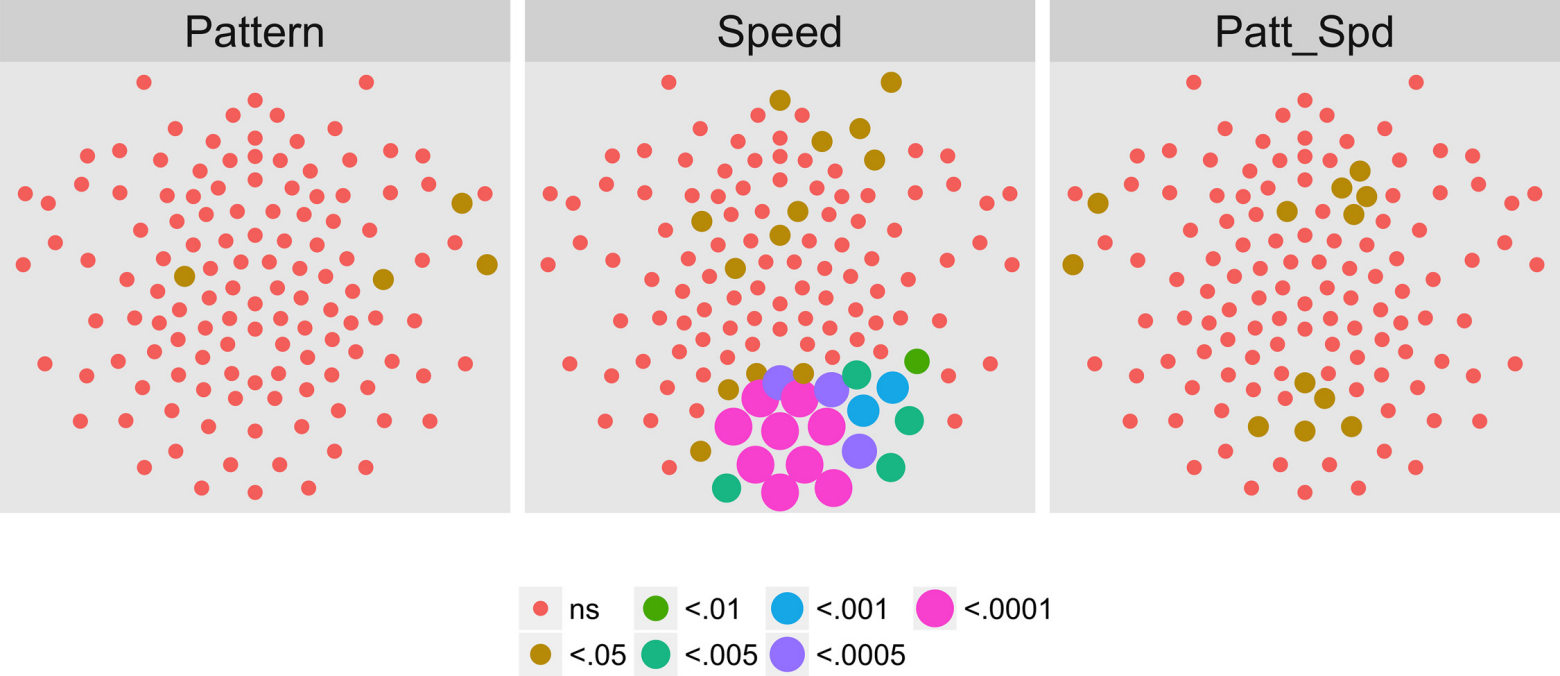
Channel-wise MANOVA on 3F1. Plots of individual channel-level effects from a MANOVA on the real and imaginary EEG components for each condition at the third harmonic (3F1) of the coherence modulation frequency (3.6 Hz). At the pre-selected threshold of *p* < .0005, responses show an effect of speed over a cluster of mid-central channel locations. Effect sizes were small to medium, with partial *η*^2^ values ranging from 0.044–0.093.

**Fig 11:**
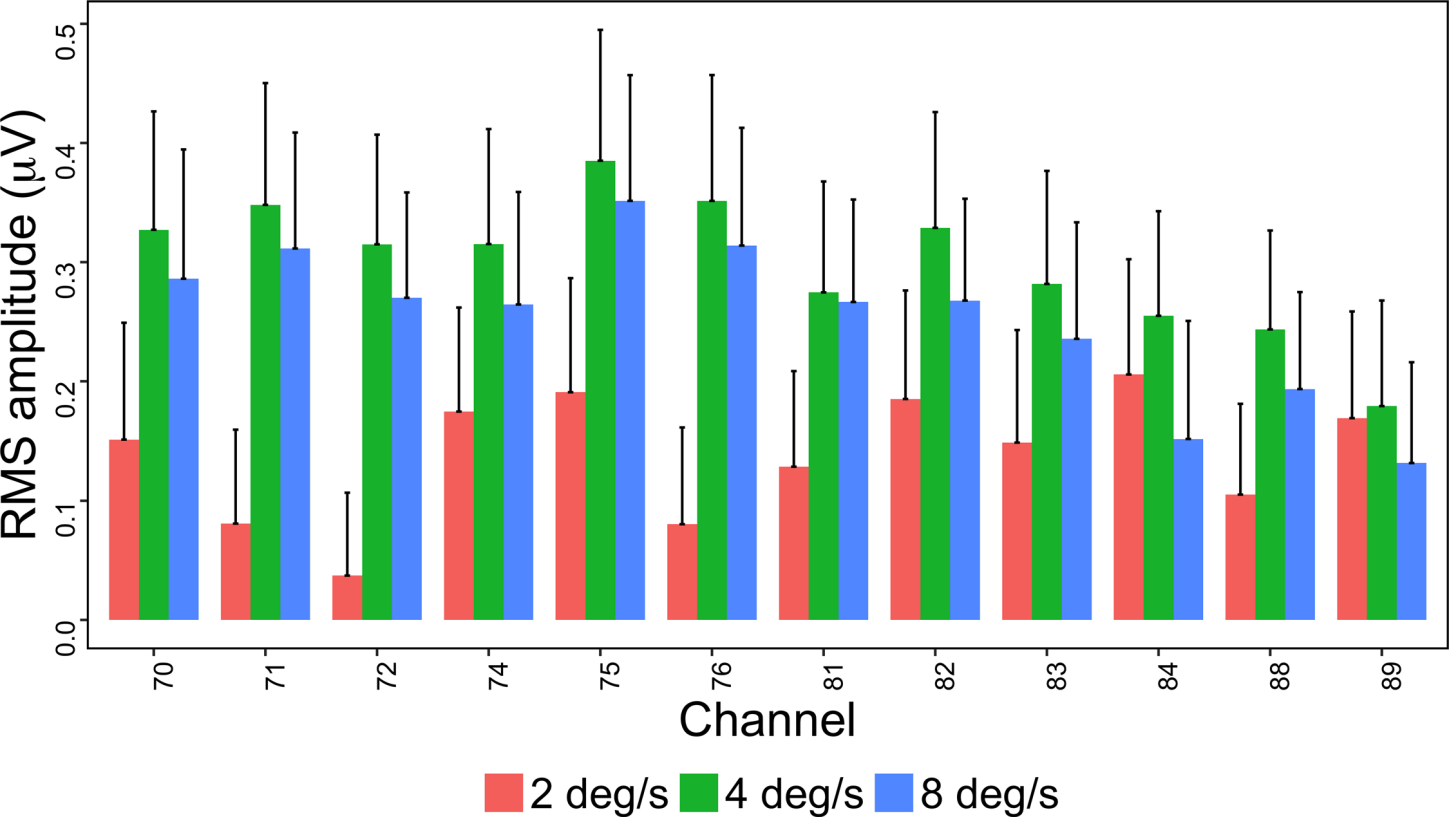
Channels With Significant Speed-Related Activity at 3F1. Plot of all channel-level pattern effects exceeding the *p* < .0005 threshold. The bars indicate the across-participants average (+ 1 SEM) of the root mean square (RMS) amplitude of the real and imaginary signal components at 3F1 (3.6 Hz) calculated for each participant. Faster speeds (4 and 8 deg/s) elicited larger responses than 2 deg/s across the majority of channels.

**Fig 12:**
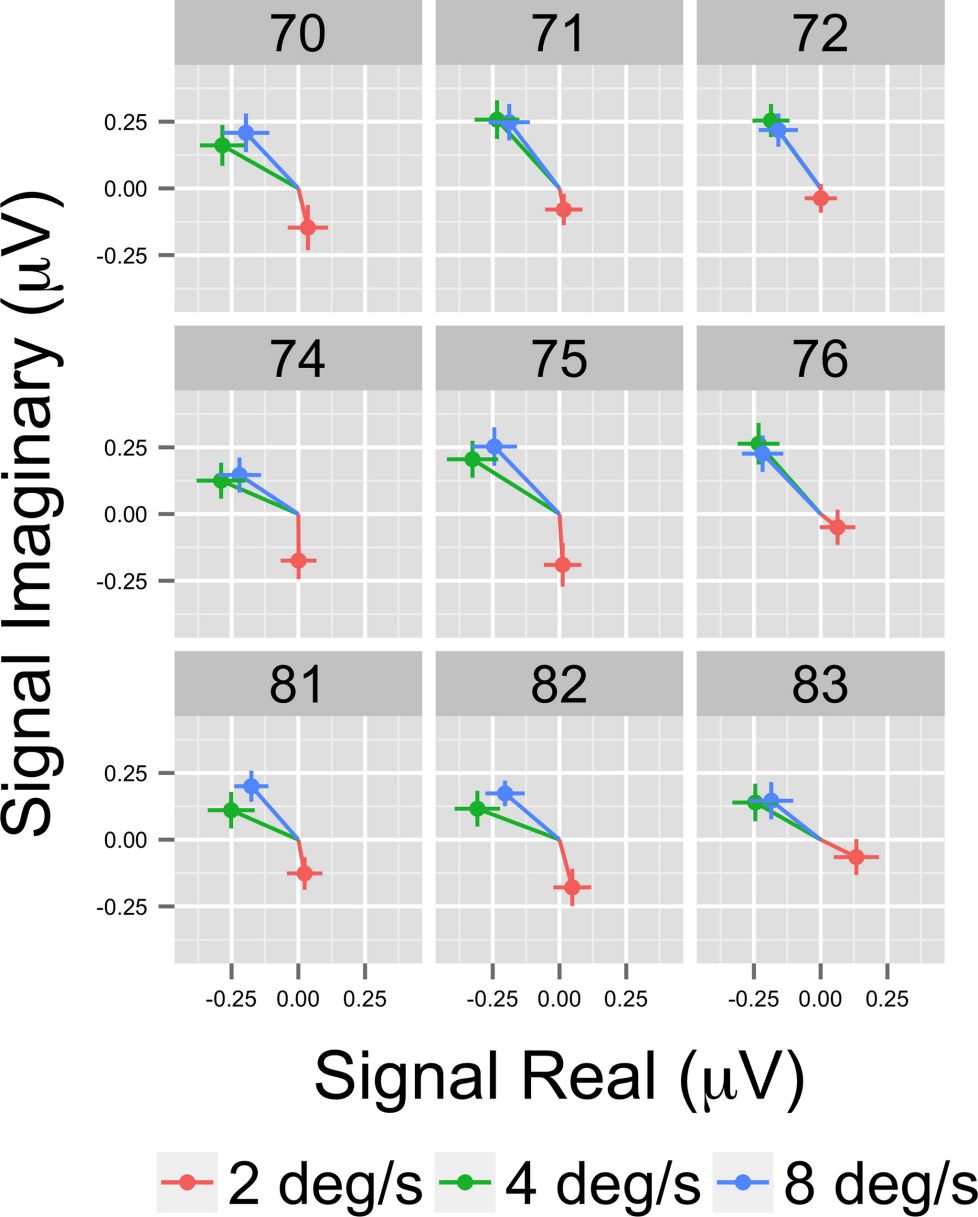
Complex Domain EEG Responses at 3F1 from Illustrative Channels. Vector plots from 9 selected channels showing the average (+ 1 SEM) real and imaginary EEG signal components.

Fig 10 shows the results of the channel-wise MANOVA. The third harmonic, like the first harmonic and other odd harmonics of the fundamental, should be sensitive either to the onset or offset of motion coherence. The figure shows a midline cluster of 12 channels with statistically significant responses to speed, but no channels that met threshold for pattern. Fig 11 shows the average amplitude by speed illustrating increased amplitudes to faster speeds, and Fig 12 depicts the complex domain results for nine representative channels. It shows that the 4 and 8 deg/s responses had similar phases to one another and these phases differed from those to 2 deg/s motion. Channel-wise statistics are reported in the Supplemental Materials (S2 Table). Three channels that met the T2Circ criterion (Fig 9) showed significant (*p* < .0005) multivariate effects for speed.

#### Responses at Dot Update Rate (1F2)

**Fig 13:**
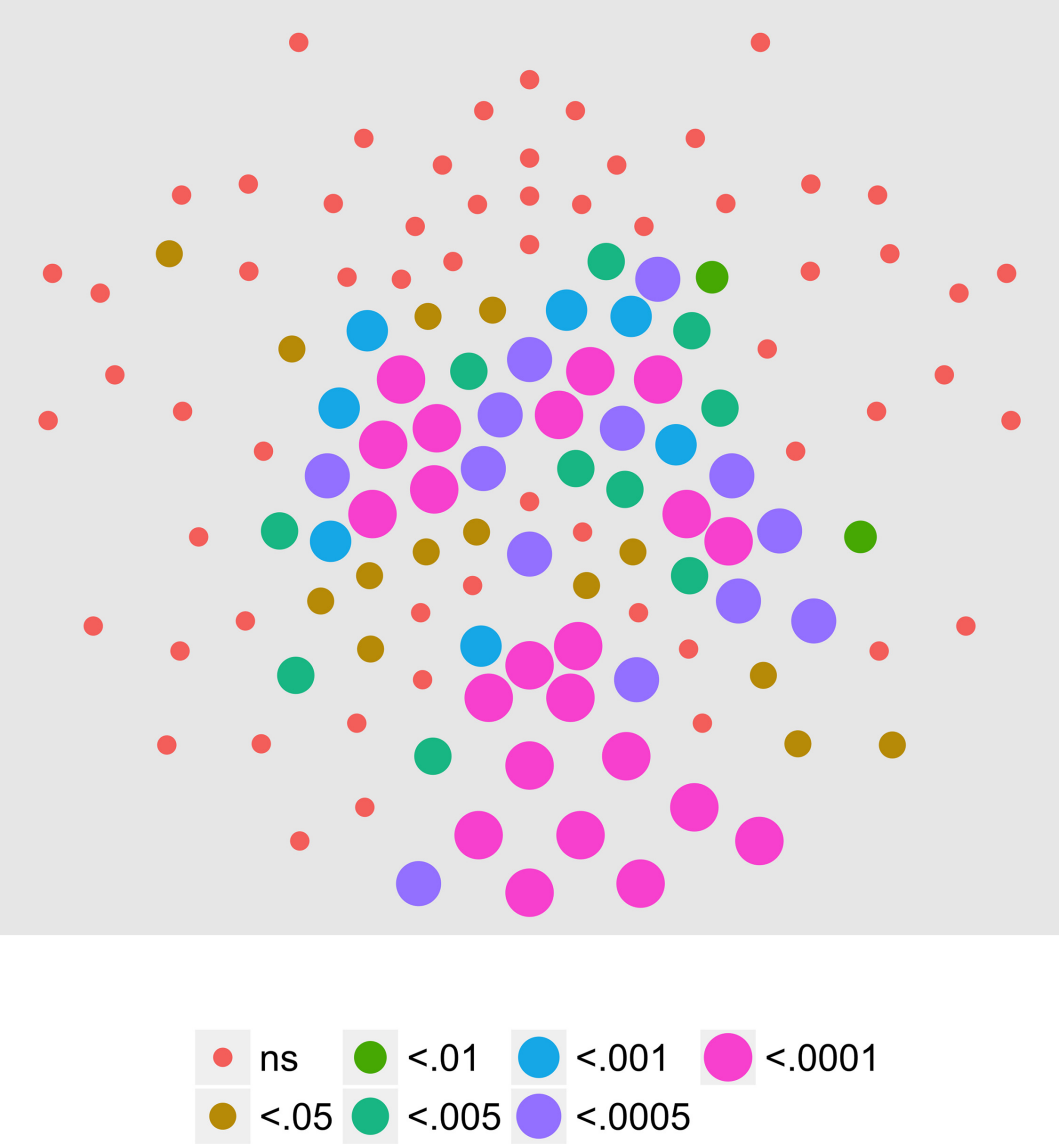
T2Circ Values by Pattern and Speed -- Children -- 1F2. Plots of individual channel-level effects from a T2Circ statistic computed on the real and imaginary EEG components at the dot update rate (1F2 or 24 Hz) for each of the three pattern (rows) and speed (columns) conditions.

Finally, we turned to an analysis of the evoked EEG responses at the dot update rate (1F2 or 24 Hz). Responses at the dot update rate have been linked to local motion and luminance changes in prior research [17, 61, 69] but not strongly to global motion patterns. The dot displacement per update cycle was equivalent across all pattern types and coherence modulations in order to isolate this local motion/luminance change signal. Fig 13 shows the results of the T2Circ analysis. Thirty-five channels met criterion grouped into a central posterior cluster and both a right and left central/anterior cluster.

**Fig 14:**
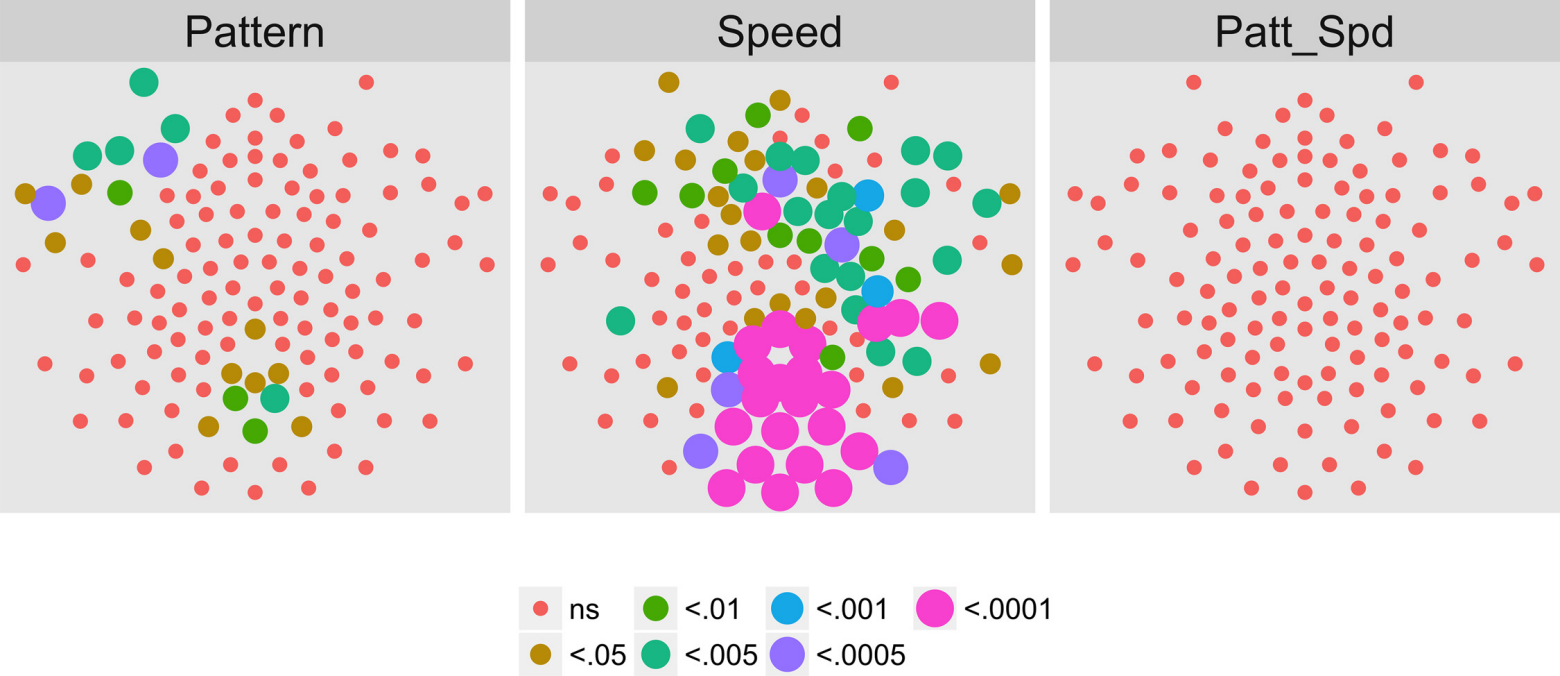
Mass Univariate MANOVA for 1F2. Plots of individual channel-level effects from a MANOVA on the real and imaginary EEG components for each condition at the dot update rate (1F2 or 24 Hz). At the pre-selected threshold of *p* < .0005, responses show a large cluster of channels with a statistically significant effect of speed over ventral and dorsal mid-central channel locations. Effect sizes were small to large, with partial *η*^2^ values ranging from 0.045–0.214. Channel-wise statistics are reported in the Supplemental Materials (S3 and S4 Tables).

Fig 14 depicts the results of the channel-wise MANOVA analysis on the 1F2 real and imaginary components. The figure reveals a large number (29) of channels showing a significant effect of speed, but only two showing a significant effect of pattern, and none showing an effect for the interaction. Most of the channels reaching criterion clustered around the posterior midline with the exception of two right frontal inferior channels. Eighteen channels that met criterion for the T2Circ test (Fig 13) also met criterion for the multivariate effect of speed.

**Fig 15:**
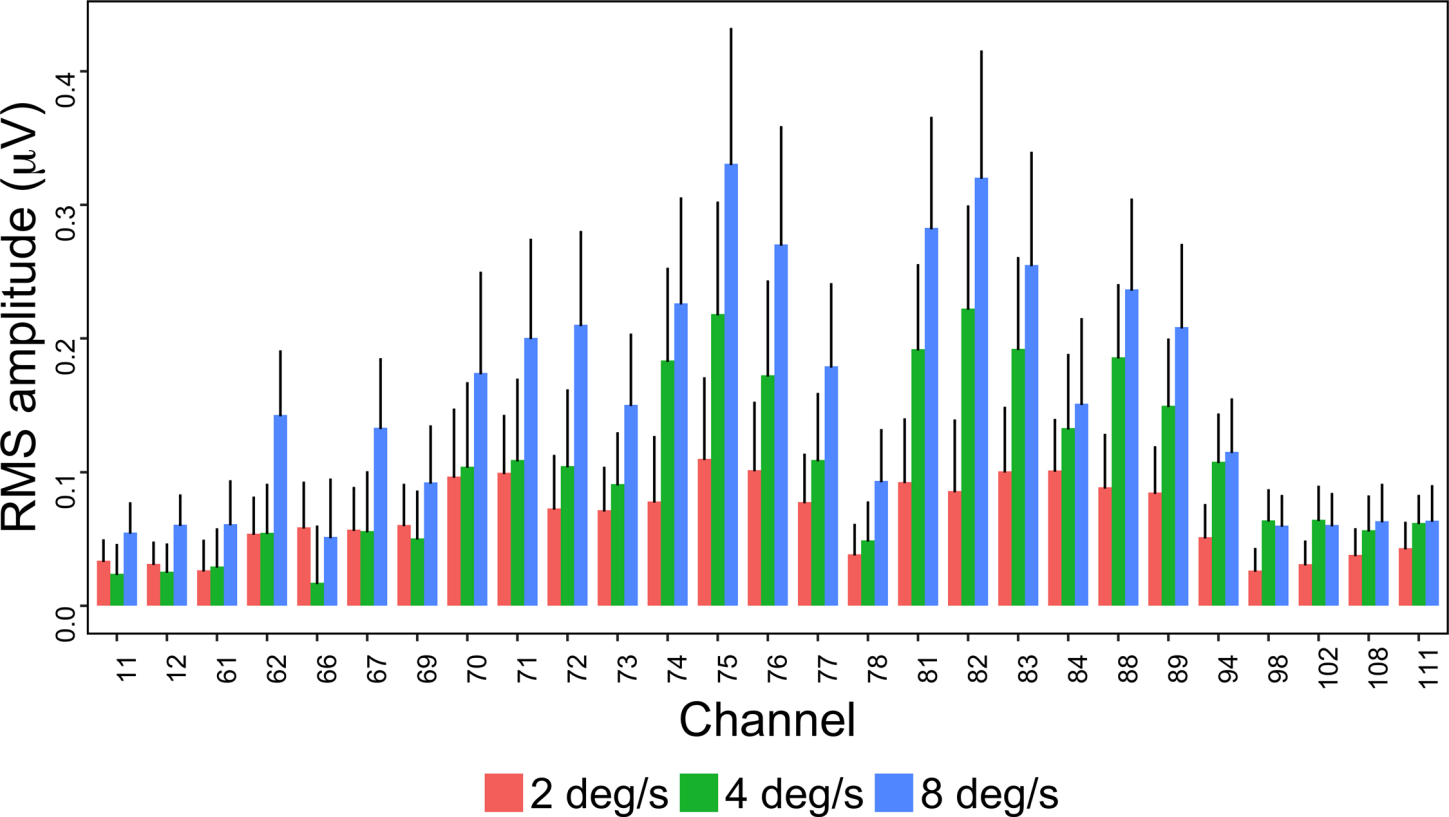
Channel-level Effects for Speed at the *p*< = .0005 Threshold for 1F2. The bars indicate the root mean square (RMS) average (+ 1 SEM) of the real and imaginary EEG signal components at 1F2 (24 Hz). Four and 8 deg/s flow patterns elicit larger responses than 2 deg/s flows.

**Fig 16:**
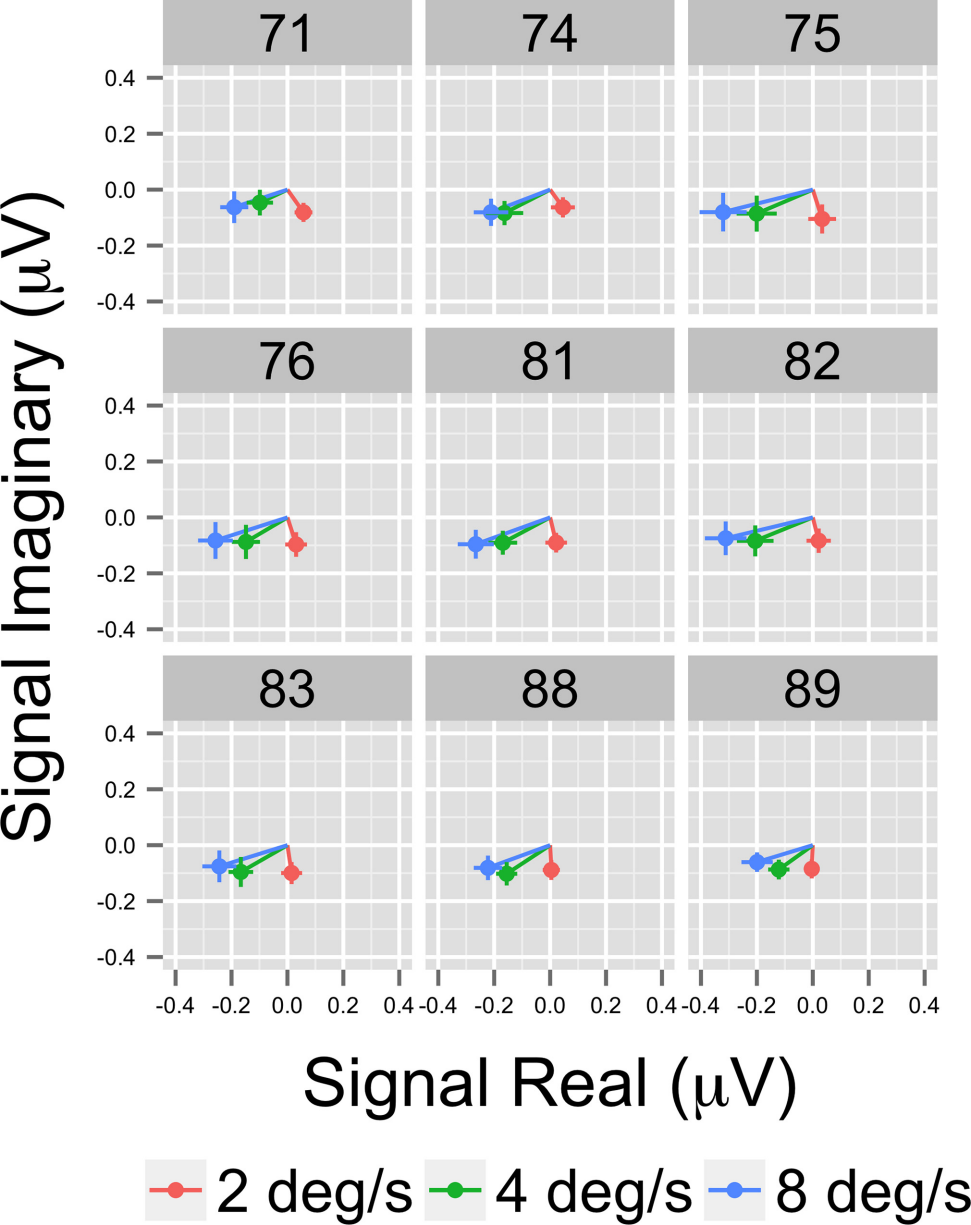
Complex Domain EEG Responses at 1F2 from Illustrative Channels. Vector plots from nine selected channels showing the average (+ 1 SEM) real and imaginary EEG signal components. Responses to the faster (4 and 8 deg/s) speeds have similar amplitudes and the relative phases are similar across the channels even though amplitudes differ.

Fig 15 indicates that the effect of speed stems from higher amplitude responses to the 4 and 8 deg/s flows relative to 2 deg/s. Fig 16 shows a vector plot of the nine highest amplitude channels. The figure confirms that the faster (4 and 8 deg/s) speed conditions show larger evoked amplitudes and that the relative phase of the responses is similar

#### Comparison With Adult Data

**Fig 17:**
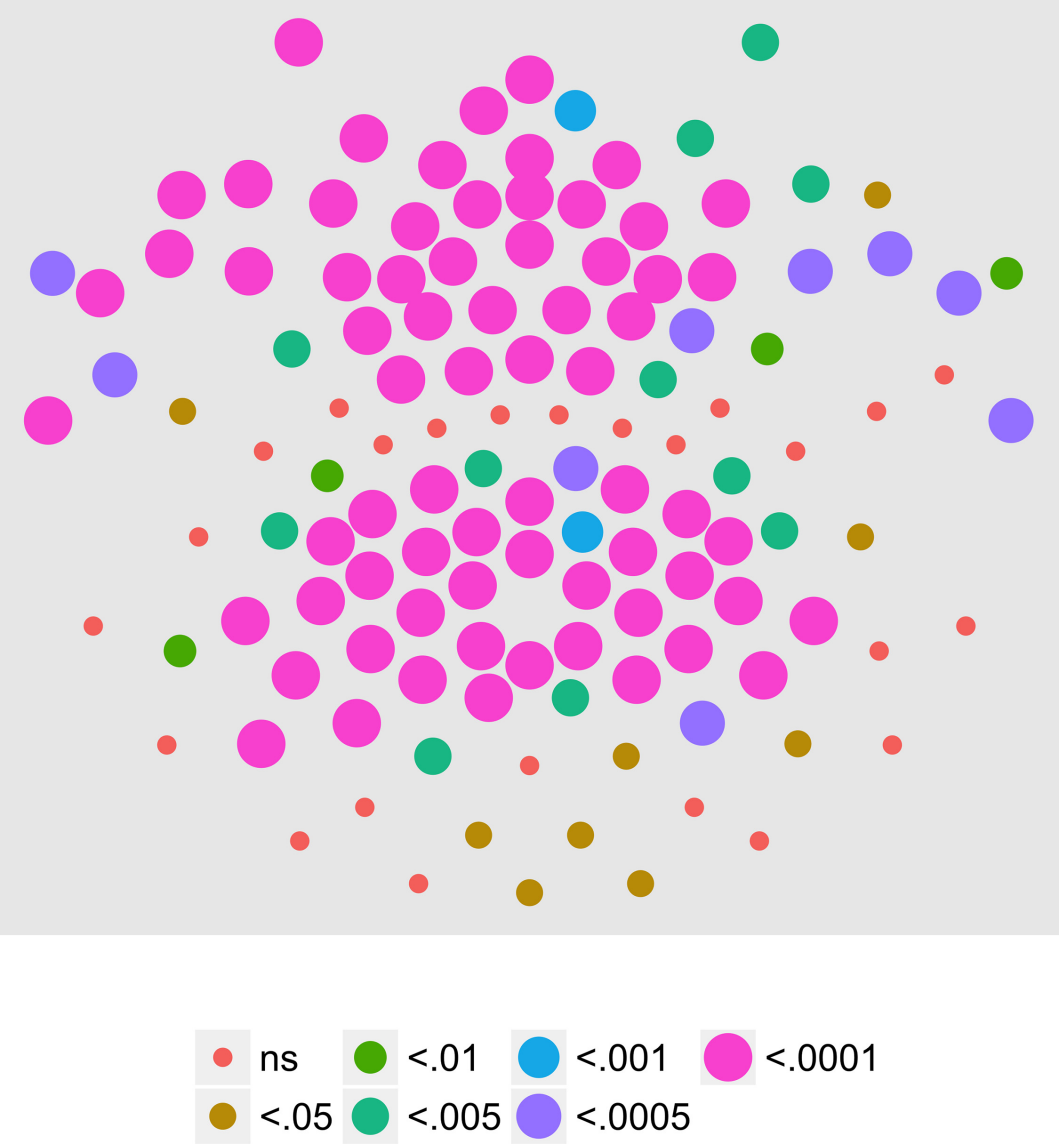
T2Circ Values by Pattern and Speed -- Adults -- 1F1. Plots of individual channel-level effects from a T2Circ statistic computed on the real and imaginary EEG components at the dot update rate (1F1 or 1.2 Hz) for each of the three pattern (rows) and speed (columns) conditions.

**Fig 18:**
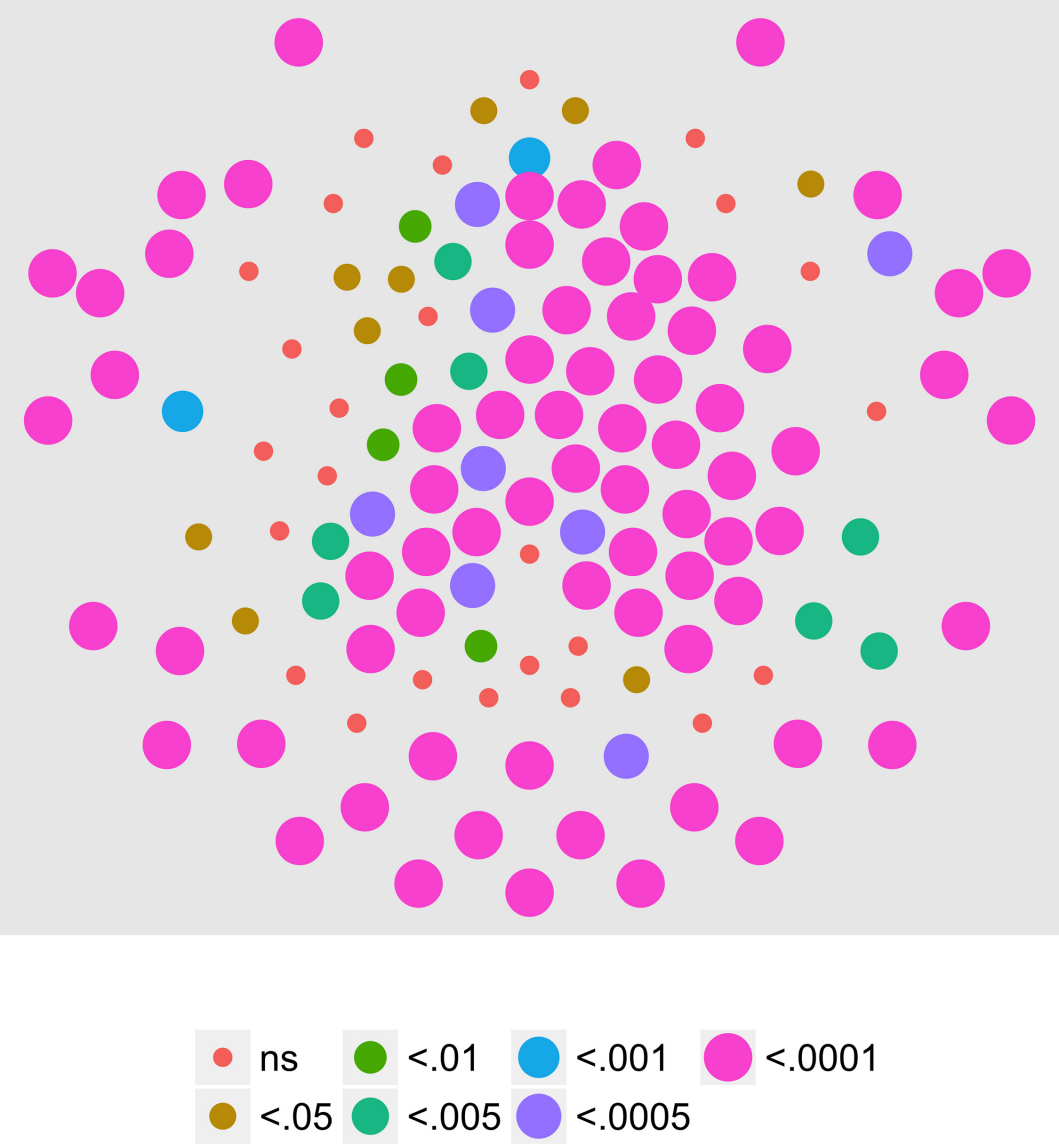
T2Circ Values by Pattern and Speed -- Adults -- 2F1. Plots of individual channel-level effects from a T2Circ statistic computed on the real and imaginary EEG components at the dot update rate (2F1 or 2.4 Hz) for each of the three pattern (rows) and speed (columns) conditions.

**Fig 19:**
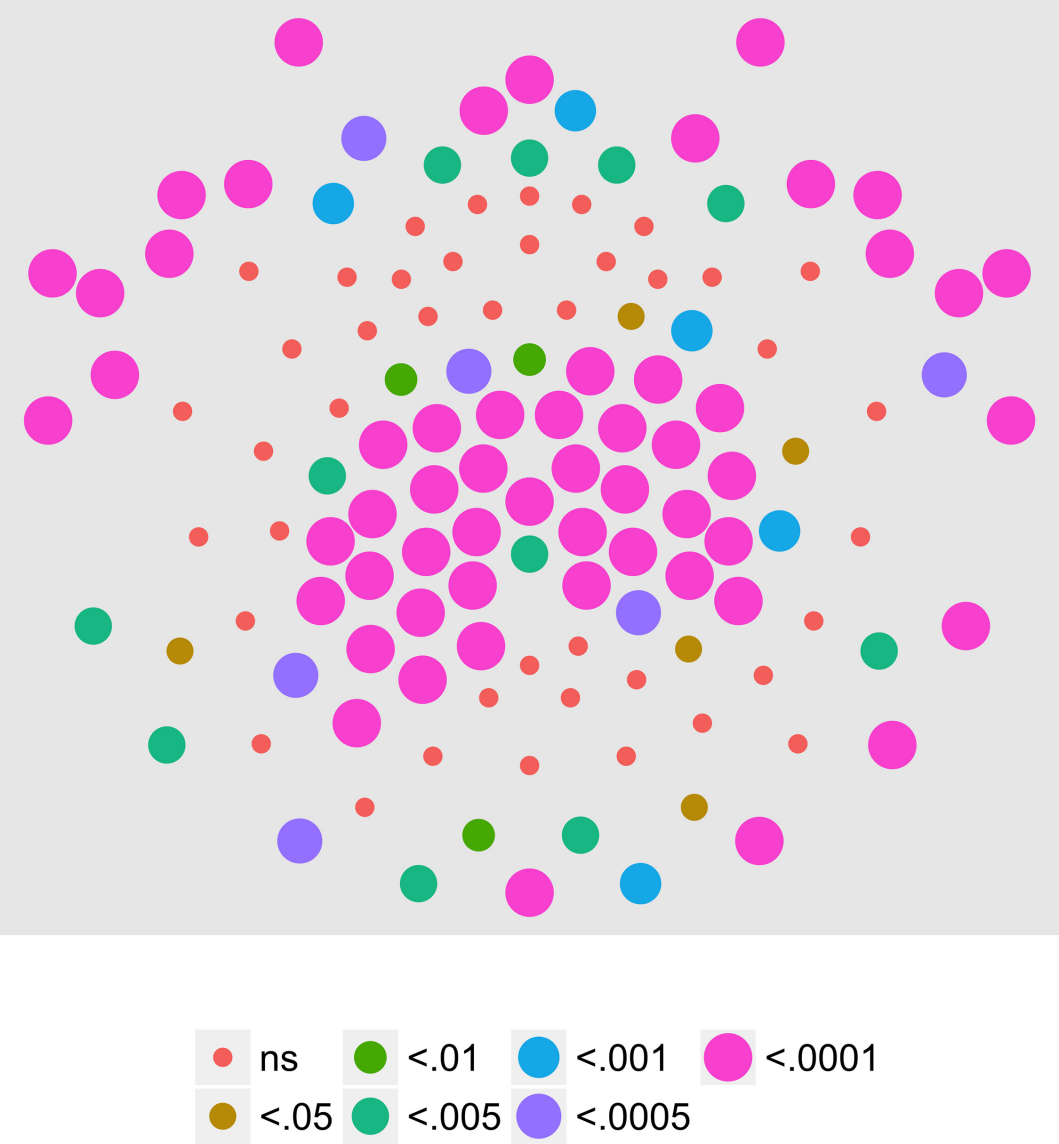
T2Circ Values by Pattern and Speed -- Adults -- 3F1. Plots of individual channel-level effects from a T2Circ statistic computed on the real and imaginary EEG components at the dot update rate (3F1 or 3.6 Hz) for each of the three pattern (rows) and speed (columns) conditions.

**Fig 20:**
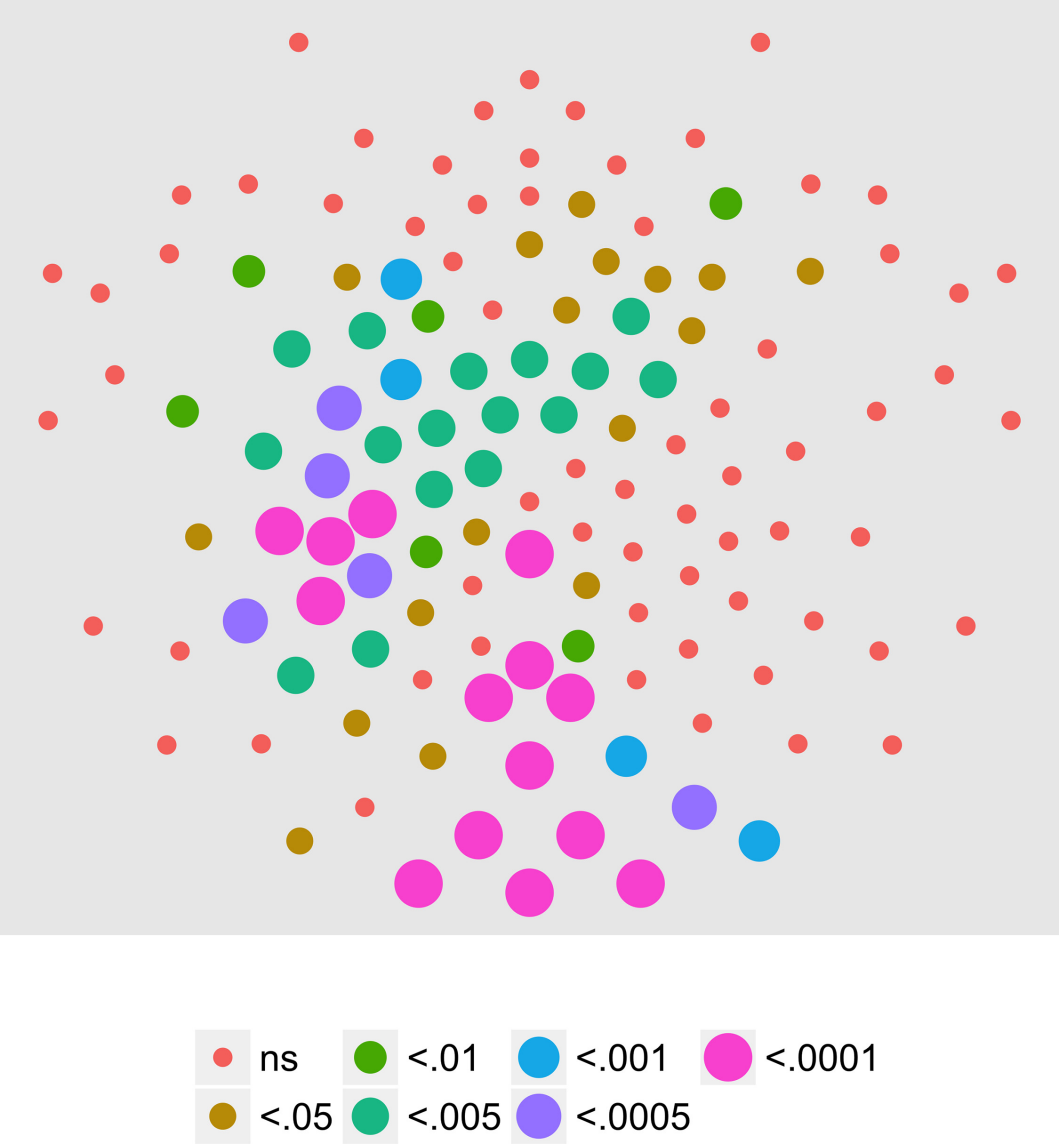
T2Circ Values by Pattern and Speed -- Adults -- 1F2. Plots of individual channel-level effects from a T2Circ statistic computed on the real and imaginary EEG components at the dot update rate (1F2 or 24 Hz) for each of the three pattern (rows) and speed (columns) conditions.

We had data available from adults who had participated in the identical experimental conditions in a previous study [17], but analyzed with a different procedure. So, we conducted an analysis on that sample identical to the one reported here. Figs 17–20 show the results of the T2Circ analysis, comparable to Figs 3, 7, 9 and 13 for the child participants. At 1F1, adults showed a widespread pattern of statistically significant channels (78 in total), with large clusters over central bilateral and frontal channels. At 2F1, 81 channels met criterion clustered in a posterior bilateral pattern and in a central anterior region. At 3F1, 19 channels meet criterion. These were clustered to the left and right of the midline over the central part of the scalp with additional channels over left frontal areas. At 1F2, 19 channels met criterion clustered over the posterior midline or left central channels. Across harmonics, adults show larger numbers of channels responsive to motion coherence modulations than do children.

**Fig 21:**
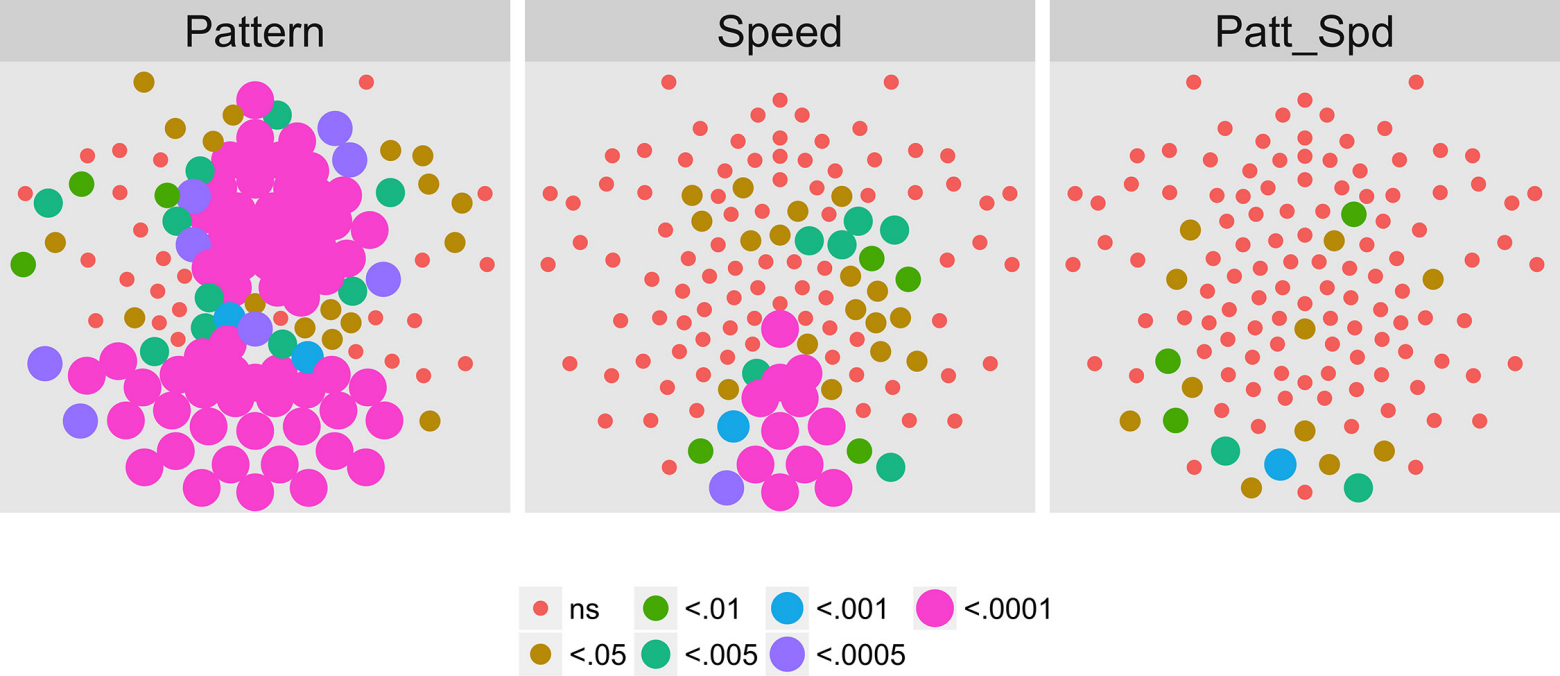
Mass Univariate MANOVA for 1F1 on Adult Data from [17]. Plots of individual channel-level effects from a MANOVA on the real and imaginary EEG components for each condition at the dot update rate (1F1 or 1.2 Hz). At the pre-selected threshold of *p* < .0005, responses show a large cluster of channels with a statistically significant effect of pattern over bilateral posterior and midline frontal channels. A smaller group responsive to speed clusters over posterior midline channels. Effect sizes were medium to large, with partial *η*^2^ values ranging from 0.074–0.1933.

**Fig 22:**
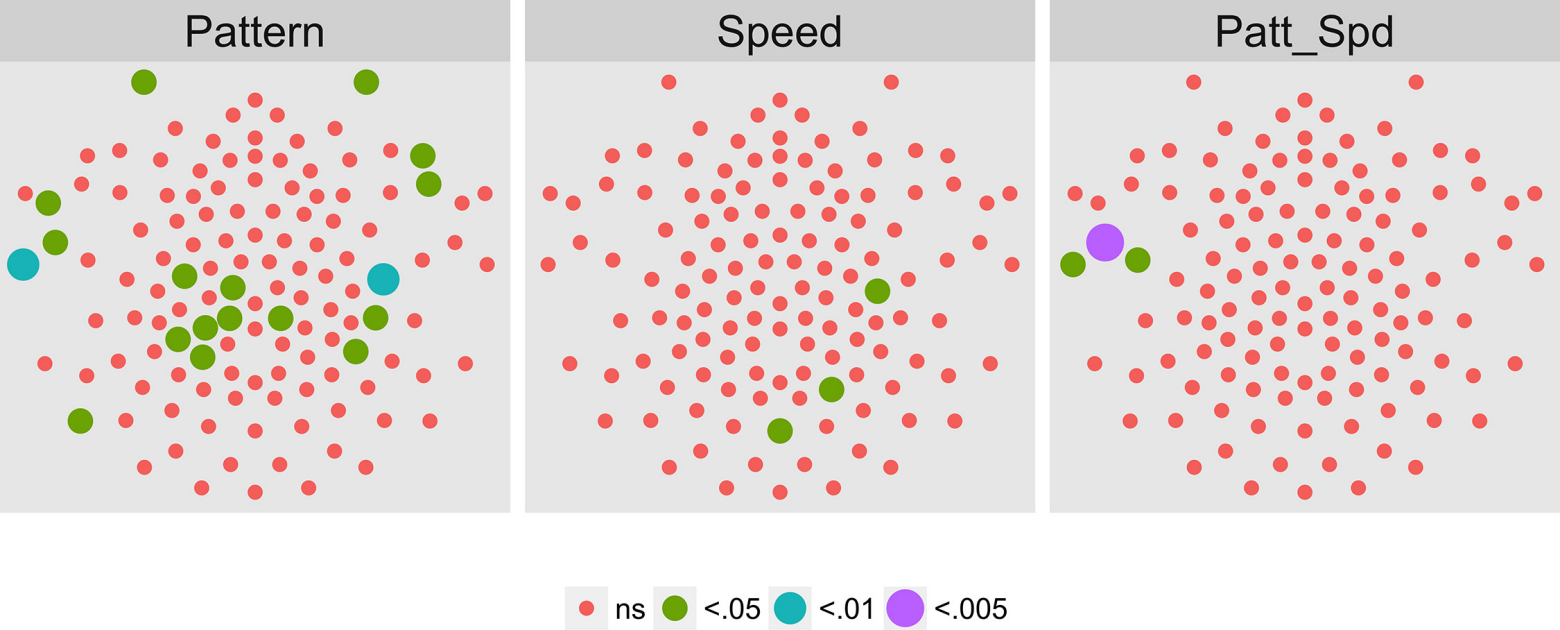
Mass Univariate MANOVA for 3F1 on Adult Data from [17]. Plots of individual channel-level effects from a MANOVA on the real and imaginary EEG components for each condition at the third harmonic of the coherence modulation frequency (2F1 or 2.4 Hz). At the pre-selected threshold of *p* < .0005 no channels met criterion.

**Fig 23:**
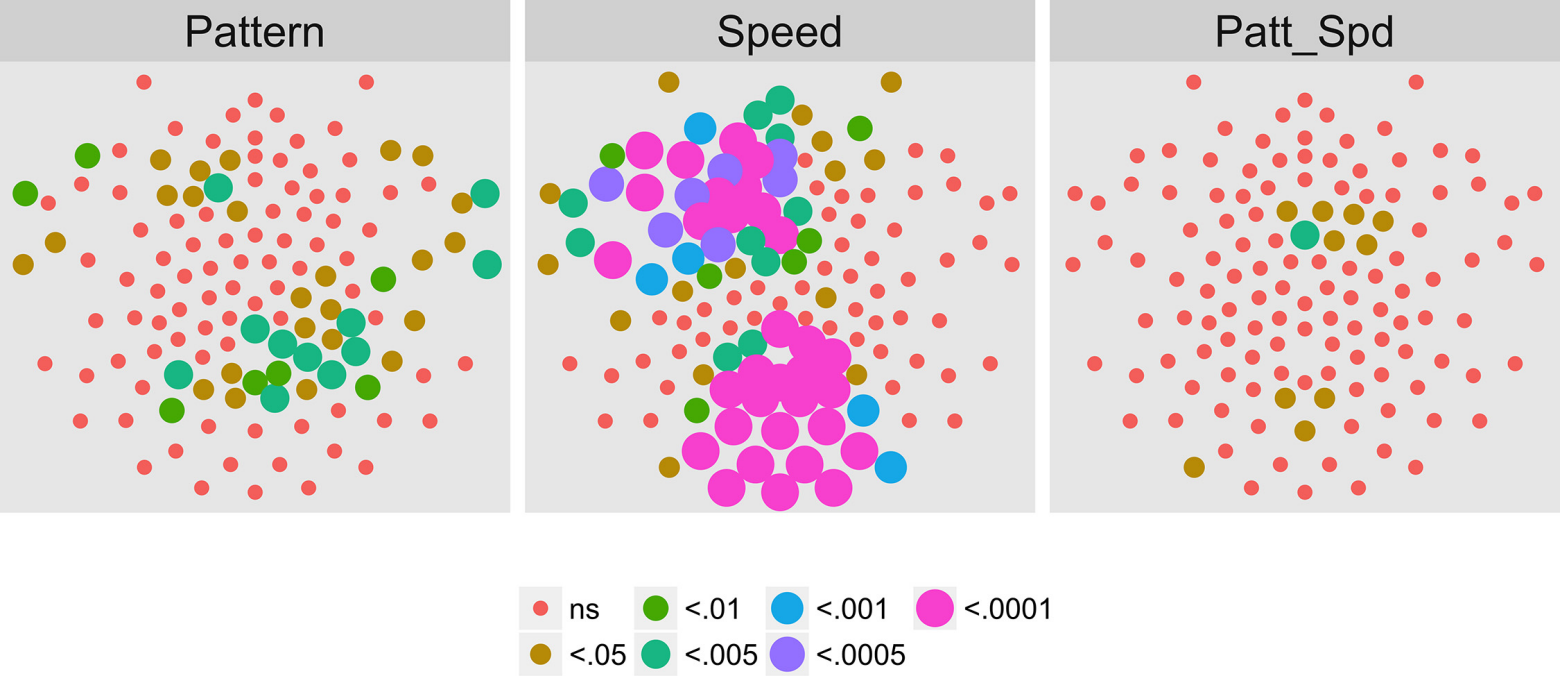
Mass Univariate MANOVA for 3F1 on Adult Data from [17]. Plots of individual channel-level effects from a MANOVA on the real and imaginary EEG components for each condition at the third harmonic of the coherence modulation frequency (3F1 or 3.6 Hz). At the pre-selected threshold of *p* < .0005, responses show a large cluster of channels with a statistically significant effect of speed over midline posterior and left frontal channels. Effect sizes were medium to large, with partial *η*^2^ values ranging from 0.067–0.265.

**Fig 24:**
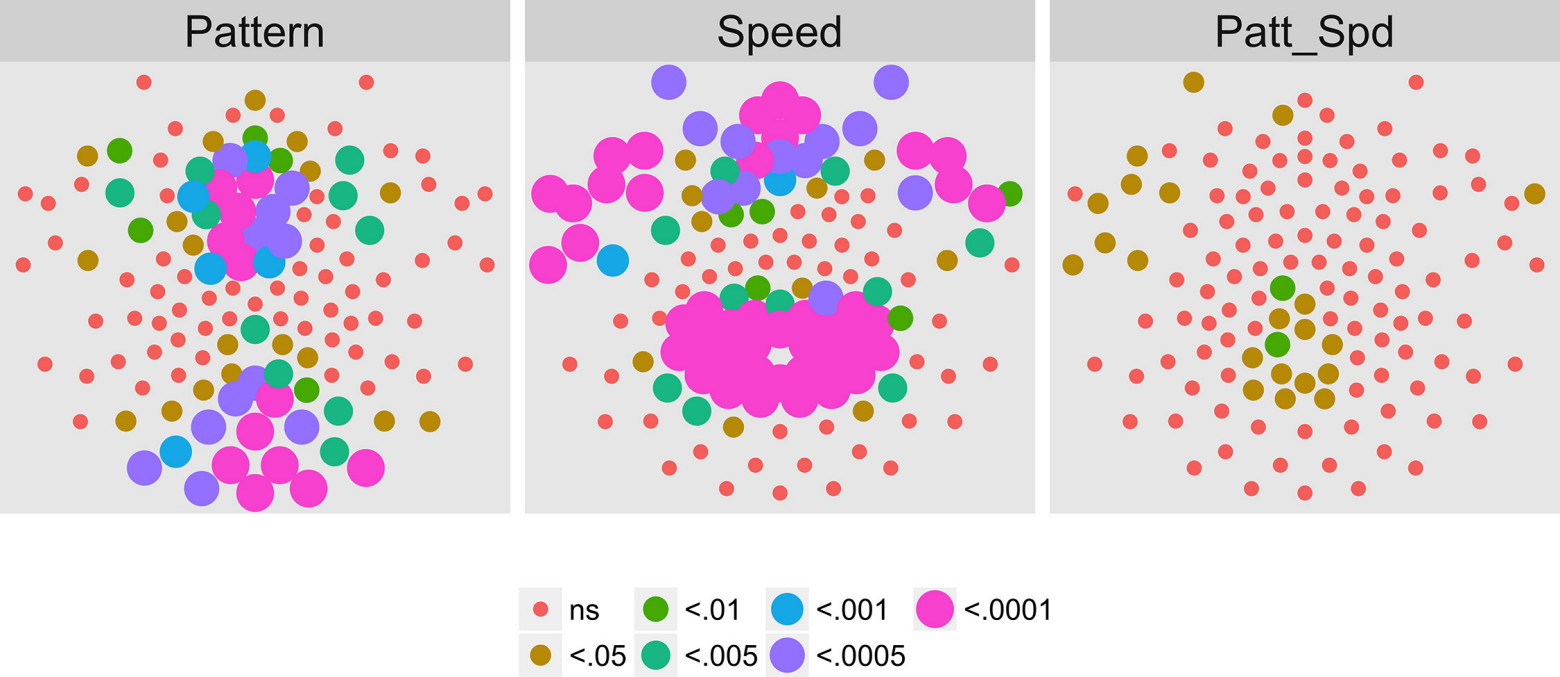
Mass Univariate MANOVA for 1F2 on Adult Data from [17]. Plots of individual channel-level effects from a MANOVA on the real and imaginary EEG components for each condition at the dot update rate (1F1 or 1.2 Hz). At the pre-selected threshold of *p* < .0005, responses show a large cluster with a statistically significant effect of speed over dorsal bilateral and frontal channels responsive and a smaller cluster of midline channels responsive to pattern differences. Effect sizes were medium to large, with partial *η*^2^ values ranging from 0.071–0.186.

Figs 21–24 show the results of a channel-wise MANOVA for the 1F1, 2F2, 3F1, and 1F2 harmonics. Fig 21 shows that adult brain responses at 1F1 consisted of widespread activity over posterior and frontal channels to differences in optic flow patterns and a midline and central cluster of channels sensitive to speed differences; 42 (out of 78) channels showing significant T2Circ activation (Fig 17) also responded to differences in flow type, most for pattern. At 2F1 adults (Fig 22) like children (Fig 8) showed no activity that met criterion. Fig 23 shows a cluster of channels responsive to speed differences at 3F1 located over midline posterior regions that do not overlap with the channels meeting the T2Circ criterion. Fig 23 shows a dorsal bilateral cluster of channels responsive to speed differences at 1F2, and a smaller cluster of midline channels responsive to pattern differences.

To summarize our results, we observed evoked brain responses to coherence modulating optic flow in children that showed more similarities to adults than to our prior [62, 64] data from infants. At 1F1, children showed fewer channels responsive to motion coherence modulations overall (Fig 3 vs. Fig 17); channels showing differential responses to pattern types in children (Fig 4) are found in posterior bilateral areas while adults showed widespread bilateral posterior activity that also distinguished among pattern types (Fig 21). Children did not show channels selective to speed at 1F1, but adults did. Children, like adults [64], demonstrated larger peak amplitude responses for radial and rotational flow than for translational flow, with selectivity for radial or rotational flow that varied by channel. Children showed channels responsive to flow pattern differences over right lateral channel locations. But, unlike adults who showed widespread bilateral activity, children showed a right unilateral cluster of activation.

At 2F1, children and adults showed similar spatial profiles of activation to motion coherence modulations across patterns and speeds (Fig 7 and Fig 18), and neither group showed channels selective for pattern or speed (Fig 8 and Fig 22).

At 3F1, children showed a few channels responsive to motion coherence overall but these differed in spread and location from adults (Fig 9 and Fig 19), however both groups showed a similar cluster of posterior channels sensitive to speed differences (Fig 10 and Fig 23).

Finally, children, like adults showed evoked activity to the dot update rate (1F2) across patterns and speeds that clustered around the posterior midline (Fig 13 and Fig 20). Children show channels selective for speed like adults (Fig 14 and Fig 23) with amplitudes that increased with increasing speed. The children's cluster of active channels over the dorsal and ventral midline is consistent with some prior findings [17, 64] from infants and adults. However, adult speed-related responses at 1F2 were grouped in a dorsal bilateral cluster, and adults showed pattern-related sensitivity at 1F2 that children did not show.

In combination, the results indicate that children show evoked global and local motion responses that are similar in a number of respects to adult observers, but with some specific differences that are likely to reflect immaturity in motion processing systems. In particular, the modulation of motion coherence evokes less widespread or robust activity in children than in adults. Children show relatively adult-like responses to coherence modulations in flow *across* pattern types and speeds, but less selective or less robust responses to flows *differing* in pattern or speed. The results also suggest that coherence-varying optic flows differing in pattern and speed activate separable components of a spatially distributed motion processing network in both children and adults.

Comparing the findings to prior work, it has been shown that the sudden onset of radial motion generates right lateral EEG activation patterns in 10 to 12 year-old children [68]. So, a right-lateralized pattern of motion-related activation may be a characteristic of immature motion processing. For example, Wattam-Bell and colleagues [22] found that 8.6 deg/s, coherence-modulating rotational flow patterns activated right lateral channels in infants. Adults in the Wattam-Bell et al. [22] study showed a midline cluster of activity, similar to the midline cluster Fesi et al. [17] found at 8 and 16 deg/s speeds. Further, both children and adults in the current study showed speed-selective EEG activity at 3F1, but only adults showed speed selectivity at 1F1. This suggests that the dynamics of cortical activity linked with global motion processing, including possibly the tagging of stimulus dimensions by frequency [76], continues to develop into late childhood and early adolescence. The cluster of EEG responses over lateral channels are likely to reflect the activity of integrative motion processing in lateral cortex such as MT/V5 and MST which may demonstrate slower and more protracted courses of development in humans and monkeys than primary visual cortex [44, 45].

The study has several limitations. We sampled a relatively children over an age range in which prior behavioral data suggest that there are changes in motion-related perceptual judgments of direction [57], coherence [56, 77], displacement [78], and speed [47, 50, 78]. We did not find changes in evoked EEG patterns as a function of age, but that does not mean brain responses to motion remain unchanged in this time period, merely that we failed to detect them. Still, evoked EEG responses to motion vary considerably even in older children [69]. In a prior study by Kubova and colleagues, 17% of 10 to 12 year-olds showed no detectable EEG response to the onset of radial motion; 23% showed no detectable response to the onset of translational motion [69]. We are unaware of developmental data that combine evoked EEG with behavioral responses to motion in children, but it is possible that behavioral measures may serve as a more sensitive index to developmental changes in certain types of motion processing. Certainly, different dimensions of motion processing appear to develop at different rates. Falkenberg and colleagues [58] found that the mature *detection* of motion emerges by age 5, but the *discrimination* of different directions of motion continues to mature into adolescence.

Moreover, some argue that characterizing development in motion sensitivity as a change in peak speed sensitivity from fast to slow—as opposed to more systematically examining evoked responses across ranges of dot displacement sizes and update rates—oversimplifies what may be a more complex pattern of change in spatial and temporal tuning to motion [44, 62, 64]. Speed is a function of change in location (space) as a function of time, so the visual system's sensitivity to detect spatial and temporal changes is important to characterize motion processing fully. Meier and Giaschi [64] assessed sensitivity to translational motion in 3–12 year olds across a wide range of spatial and temporal offsets. They found a range of intermediate speeds (4–12 deg/s) where relative maturity depended on the specific spatial and temporal offsets of the displays. In the current study, the temporal offset (1/update rate or 1F2) was fixed at 1/24 or 41.6 ms and the speeds were 2, 4, and 8 deg/s. In the Meier and Giaschi data, peak motion sensitivity in children was in 1–8 deg/s at the 50 ms temporal offset most comparable to the offset used in the current study. At the shorter, 17 ms, temporal offset children's sensitivity curve shifted to fast (> 15–20 deg/s) speeds [64]. Thus, the current results are comparable to Meier and Giaschi [64] when stimulus parameters are considered in detail. This illustrates the importance of examining display parameters carefully in comparing findings across studies, especially when the studies might appear contradictory [47].

The current child data showed a finding prior adult data [17] did not show using a different, somewhat less sensitive, analysis pipeline—statistically significant activation to the dot update rate over a cluster of left inferior frontal channels [17]. Under the new analysis, however, we find that both children and adults show activity at the update rate over inferior frontal channels. Rather than representing activity in some frontal locus, this may instead reflect activity of a strong, directionally oriented cortical dipole located elsewhere in the brain, such as the posterior cortex. Coherently activating spatially contiguous regions of neural tissue can create electrical dipoles whose activity can be measured at distant scalp locations. The eyes, for example, are large, moveable electrical dipoles whose activity may occasionally be observed over posterior EEG channels during eye movements or large changes in luminance. EEG has poor spatial resolution so functional magnetic resonance imaging studies would be needed to confirm the spatial locus of activation to the time-varying flow patterns used in the current study.

Another limitation concerns the lack of concurrent behavioral data. The results reflect brain responses to optic flow during active fixation on the screen, a state commonly evoked in fMRI studies with pediatric populations [79, 80]. An experimenter monitored participant attention and arousal during the task, but it is possible that both varied across the time course of the session and between participants. On the other hand, the use of passive observation enables these results to be readily compared with prior infant [22, 62, 64–67] and adult studies [17, 22, 64, 66] using similar techniques and displays. Nevertheless, by assessing flow related activity in an active fixation state, our results may underestimate the strength of response as it occurs in more attention-demanding circumstances or when the flow patterns are relevant for other behaviors.

Finally, an observer monitored eye position, but we did not record it. Still, to account for the observed SSVEP responses, the eyes would have to move in a consistent, phase-locked manner to the onset and offset of coherent motion. This seems unlikely, especially for the radial and rotational flow patterns that showed the strongest evoked effects because these patterns have weak direction signals. Plus, the frequency of direction shifts (in/out; left/right) is half (0.6 Hz) the pattern modulation frequency (1.2 Hz) we analyzed in detail. For these reasons, we think it is unlikely that eye movements substantially contaminate or systematically explain our results.

While focused on the question of brain activation to largely abstract flow patterns, there are important real-world implications for understanding the development of brain responses to varying optic flow patterns and speeds [81]. In natural conditions, the eyes and head scan the environment, yielding motion patterns on the retina that are a complex mixture of radial, rotation, translation, and shear, varying in speed as a function of distance from the observer and the speed of observer movement [60]. So, in natural conditions, the visual brain is likely confronted with the task of parsing a complex mixture of motion types. Indeed, recent evidence suggests that the statistics of optic flow experienced by infant and child observers changes over the course of development [60, 61, 81] due, in part, to changes in body posture and dimensions [81]. Successful navigation requires the accurate perception of objects, surfaces, and patterns of movement in the environment [1, 82] in order to planning and execute locomotion, catching and throwing, and road-crossing actions [55, 83–89], among others. How developing brain activity contributes to the use of optic flow for action planning remains an open question although the integration of multisensory cues in navigational tasks occurs over a prolonged developmental time course [90]. Future studies should explore the relationship between changes in brain activity to complex motion and the changes in performance on a variety of optic flow-dependent skills. Moreover, functional imaging methods with higher spatial resolution should be employed to provide more precise information about the localization of optic flow related responses.

## Conclusions

This study demonstrates that evoked patterns of brain activity to temporally changing optic flow patterns in children show many similarities to adults. Children, like adults, show clusters of brain activity that distinguish among different patterns and speeds of optic flow. Channels that distinguish flow patterns and speeds do so in EEG amplitudes, phases, or both. Radial and rotational flow tends to evoke larger amplitudes and different phase profiles than translational flow. Pattern-sensitive responses arise over lateral scalp locations likely to overlay motion sensitive cortical regions hMT and hMST while speed-sensitive responses cluster around the midline. Children's responses are more focal and more strongly lateralized than adults. However, children and adults show local motion and luminance responses that cluster around the midline and which increase with increasing speed. Taken together the results imply that motion processing networks undergo substantial change between infancy and early childhood but continue to undergo fine-tuning throughout the transition to adulthood.

## Supporting Information

S1 Table Statistics for Children and Adults for Channels Meeting Criterion at 1F1 for Pattern. MANOVA results from children (left columns) and adults (right columns); only channels meeting the *p* < .0005 criterion are shown.
(PDF)

S2 Table Statistics for Children and Adults for Channels Meeting Criterion at 3F1 for Speed. MANOVA results from children (left columns) and adults (right columns); only channels meeting the *p* < .0005 criterion are shown.
(PDF)

S3 Table Statistics for Children and Adults for Channels Meeting Criterion at 1F2 for Pattern. MANOVA results from children (left columns) and adults (right columns); only channels meeting the *p* < .0005 criterion are shown.
(PDF)

S4 Table Statistics for Children and Adults for Channels Meeting Criterion at 1F2 for Speed. MANOVA results from children (left columns) and adults (right columns); only channels meeting the *p* < .0005 criterion are shown.
(PDF)
