# Supplementary material for: Children's Brain Responses to Optic Flow Vary by Pattern Type and Motion Speed
Source: PLoS One. 2016 Jun 21;11(6):e0157911. doi: 10.1371/journal.pone.0157911 (PMC4915671; doi:10.1371/journal.pone.0157911)
Supplement: S1 Table — MANOVA results from children (left columns) and adults (right columns); only channels meeting the p < .0005 criterion are shown. (PDF) [file pone.0157911.s001.pdf]

Statistics for Children and Adults for Channels Meeting Criterion at 1F1 for Pattern

	Children					Adults				
Channel	*F*	Df1	Df2	*p*	partial η^2	*F*	Df1	Df2	*p*	partial η^2
2						5.615315	4	304	0.0002257	0.0688022
3						7.733117	4	304	0.000006	0.0923543
4						12.815081	4	304	0	0.1442895
5						13.799209	4	304	0	0.1536674
6						16.730596	4	304	0	0.1804215
7						12.984161	4	304	0	0.1459154
8						5.357861	4	304	0.0003505	0.0658555
9						9.299713	4	304	0.0000004	0.109024
10						7.955931	4	304	0.0000041	0.0947632
11						11.694167	4	304	0	0.1333517
12						11.659692	4	304	0	0.1330109
13						11.860705	4	304	0	0.1349944
15						8.73884	4	304	0.0000011	0.1031267
16						7.705604	4	304	0.0000063	0.092056
17						6.344807	4	304	0.0000647	0.0770517
18						6.609145	4	304	0.0000412	0.080005
19						8.141944	4	304	0.000003	0.0967644
20						8.262191	4	304	0.0000024	0.0980534
24						5.432771	4	304	0.0003084	0.0667148
29						5.675568	4	304	0.0002036	0.0694892
30	6.056696	4	448	0.000094	0.0513033	6.807185	4	304	0.0000293	0.0822052
31						6.251651	4	304	0.0000759	0.0760064
50						8.86934	4	304	0.0000009	0.1045058
56						5.471891	4	304	0.0002884	0.0671629
57						8.180265	4	304	0.0000028	0.0971756

58						13.231699	4	304	0	0.1482847
59						6.528068	4	304	0.0000473	0.0791012
60						6.509217	4	304	0.0000489	0.0788908
61						6.722419	4	304	0.0000339	0.0812648
62						5.848008	4	304	0.0001515	0.0714496
63						5.96975	4	304	0.000123	0.0728287
64						11.982413	4	304	0	0.136191
65						12.081974	4	304	0	0.1371674
66						11.576635	4	304	0	0.1321886
67						6.519274	4	304	0.000048	0.079003
68						11.430213	4	304	0	0.1307353
69	5.4486	4	448	0.0002726	0.0463914	16.653773	4	304	0	0.179742
70						15.5299	4	304	0	0.1696702
71						12.537177	4	304	0	0.1416035
72						9.907484	4	304	0.0000002	0.1153274
73						14.811136	4	304	0	0.1630982
74						18.840223	4	304	0	0.1986522
75						18.732283	4	304	0	0.1977392
76						12.416644	4	304	0	0.1404333
77						7.349573	4	304	0.0000116	0.0881777
80						8.972208	4	304	0.0000007	0.1055899
81						15.651354	4	304	0	0.1707706
82						17.075279	4	304	0	0.1834566
83						15.435049	4	304	0	0.1688089
84						9.045861	4	304	0.0000006	0.1063645
87						9.598642	4	304	0.0000003	0.1121354
88						16.972491	4	304	0	0.1825539
89	5.71067	4	448	0.0001724	0.0485145	16.96623	4	304	0	0.1824988

90	6.350583	4	448	0.0000561	0.0536591	14.263992	4	304	0	0.1580253
91						7.48045	4	304	0.0000093	0.0896072
94	5.848806	4	448	0.0001353	0.0496297	16.097227	4	304	0	0.1747851
95	8.369864	4	448	0.0000016	0.0695345	11.83353	4	304	0	0.1347268
96	7.347214	4	448	0.0000097	0.0615617	8.885741	4	304	0.0000008	0.1046788
100	7.635749	4	448	0.0000059	0.063825					
101	7.240033	4	448	0.0000118	0.0607181					
104						8.657183	4	304	0.0000013	0.1022616
105						9.062504	4	304	0.0000006	0.1065393
106	6.665625	4	448	0.0000323	0.0561715	18.030204	4	304	0	0.1917491
107	5.67406	4	448	0.0001838	0.0482184					
109						5.565365	4	304	0.0002458	0.068232
110						7.926827	4	304	0.0000043	0.0944493
111						14.543885	4	304	0	0.160628
112						20.683486	4	304	0	0.2139299
116						7.735107	4	304	0.000006	0.0923759
117						11.685708	4	304	0	0.1332681
118						14.319002	4	304	0	0.1585381
123						9.467739	4	304	0.0000003	0.1107756
124						11.423577	4	304	0	0.1306693
