# Supplementary material for: Children's Brain Responses to Optic Flow Vary by Pattern Type and Motion Speed
Source: PLoS One. 2016 Jun 21;11(6):e0157911. doi: 10.1371/journal.pone.0157911 (PMC4915671; doi:10.1371/journal.pone.0157911)
Supplement: S3 Table — MANOVA results from children (left columns) and adults (right columns); only channels meeting the p < .0005 criterion are shown. (PDF) [file pone.0157911.s003.pdf]

Statistics for Children and Adults for Channels Meeting Criterion at 1F2 for Pattern

	Children					Adults				
Channel	*F*	Df1	Df2	*p*	partial η^2	*F*	Df1	Df2	*p*	partial η^2
4						5.41814	4	272	3.27E-04	0.0737984
5						5.774764	4	272	1.79E-04	0.0782756
6						5.280887	4	272	4.13E-04	0.0720636
7						6.758034	4	272	3.37E-05	0.0903988
11						6.69827	4	272	3.73E-05	0.089671
12						6.705412	4	272	3.68E-05	0.089758
13						6.365603	4	272	6.55E-05	0.0855988
18						5.968837	4	272	1.29E-04	0.0806939
19						7.692172	4	272	6.90E-06	0.1016244
26	5.257394	4	432	0.0003834	0.0464199					
43	5.230217	4	432	0.0004019	0.046191					
68						5.808759	4	272	1.69E-04	0.0787001
70						5.466109	4	272	3.02E-04	0.0744031
71						5.51668	4	272	0.0002768	0.0750398
72						5.273399	4	272	0.0004183	0.0719688
73						5.585293	4	272	0.0002464	0.0759023
74						8.677656	4	272	0.0000013	0.1131706
75						8.42129	4	272	0.000002	0.1101956
76						7.332515	4	272	0.0000127	0.0973353
81						8.176086	4	272	0.0000031	0.1073314
82						7.232552	4	272	0.0000151	0.0961359
83						5.635555	4	272	0.0002262	0.0765331
88						6.344983	4	272	0.0000679	0.0853451
94						6.266195	4	272	0.0000776	0.0843748
112						5.203469	4	272	0.0004709	0.0710823
