# Supplementary material for: Children's Brain Responses to Optic Flow Vary by Pattern Type and Motion Speed
Source: PLoS One. 2016 Jun 21;11(6):e0157911. doi: 10.1371/journal.pone.0157911 (PMC4915671; doi:10.1371/journal.pone.0157911)
Supplement: S4 Table — MANOVA results from children (left columns) and adults (right columns); only channels meeting the p < .0005 criterion are shown. (PDF) [file pone.0157911.s004.pdf]

Statistics for Children and Adult for Channels Meeting Criterion at 1F2 for Speed

	Children					Adults				
Channel	*F*	Df1	Df2	*p*	partial η^2	F	Df1	Df2	*p*	partial η^2
1						9.640914	4	272	3.00E-07	0.1241731
8						5.186177	4	272	4.85E-04	0.0708628
9						5.246365	4	272	4.38E-04	0.0716263
10						6.049259	4	272	1.12E-04	0.0816924
11	5.938706	4	432	0.0001167	0.0521219					
12	6.402184	4	432	0.0000518	0.0559621					
14						7.163449	4	272	1.70E-05	0.095305
15						8.547443	4	272	1.60E-06	0.111662
16						6.020174	4	272	1.18E-04	0.0813315
17						9.025074	4	272	7.00E-07	0.1171706
18						7.491067	4	272	9.70E-06	0.0992312
19						5.876617	4	272	1.50E-04	0.0795464
21						8.235776	4	272	2.80E-06	0.1080303
22						5.272048	4	272	0.0004192	0.0719517
24						5.463296	4	272	0.000303	0.0743677
25						5.200775	4	272	0.0004731	0.0710481
32						9.295594	4	272	0.0000005	0.1202603
33						7.479516	4	272	0.0000099	0.0990933
38						11.770525	4	272	0	0.1475548
42						7.282922	4	272	0.0000139	0.0967407
43						11.445036	4	272	0	0.1440623
44						7.32978	4	272	0.0000128	0.0973026
47						6.180325	4	272	0.0000897	0.0833149
48						7.318111	4	272	0.0000131	0.0971627
49						8.24668	4	272	0.0000027	0.1081579

51						10.208156	4	272	0.0000001	0.1305255
52						10.864346	4	272	0	0.1377599
53						12.645296	4	272	0	0.1568014
54						9.041858	4	272	0.0000007	0.1173629
59						14.873364	4	272	0	0.179471
60						15.500674	4	272	0	0.1856353
61	11.827816	4	432	0	0.0987068	12.318195	4	272	0	0.1533674
62	19.976994	4	432	0	0.1560983	12.256914	4	272	0	0.152721
66	5.600357	4	432	0.0002108	0.0492988	13.010975	4	272	0	0.1606076
67	17.81414	4	432	0	0.1415909	11.725745	4	272	0	0.147076
69	5.140958	4	432	0.0004695	0.0454385					
70	16.071403	4	432	0	0.1295335					
71	23.771294	4	432	0	0.1803981	9.182434	4	272	0.0000006	0.1189705
72	24.639345	4	432	0	0.185762	10.331382	4	272	0.0000001	0.1318933
73	11.14612	4	432	0	0.09355					
74	18.986432	4	432	0	0.1495154					
75	29.363026	4	432	0	0.2137622					
76	27.712499	4	432	0	0.2042	7.379967	4	272	0.0000118	0.0979036
77	20.241433	4	432	0	0.1578385	8.76466	4	272	0.0000011	0.1141757
78	11.757993	4	432	0	0.0981813	14.132253	4	272	0	0.172067
79						6.135533	4	272	0.0000968	0.082761
81	28.966627	4	432	0	0.2114867					
82	29.314125	4	432	0	0.2134822					
83	22.012762	4	432	0	0.1693123					
84	9.068068	4	432	0.0000005	0.0774598	7.90014	4	272	0.0000049	0.104086
85						11.103442	4	272	0	0.1403661
86						12.212622	4	272	0	0.1522531
87						5.280812	4	272	0.000413	0.0720627

88	25.379444	4	432	0	0.19028					
89	11.63538	4	432	0	0.097257					
91						9.108157	4	272	0.0000006	0.1181218
92						12.733018	4	272	0	0.1577176
93						8.756599	4	272	0.0000012	0.1140827
94	5.987799	4	432	0.0001071	0.0525302					
97						9.938348	4	272	0.0000002	0.1275155
98	6.337984	4	432	0.000058	0.055432	7.691789	4	272	0.0000069	0.1016199
102	6.067078	4	432	0.0000932	0.0531887					
108	6.840717	4	432	0.000024	0.059567					
111	5.736462	4	432	0.0001662	0.0504364					
120						7.145912	4	272	0.0000175	0.0950938
121						7.300296	4	272	0.0000134	0.0969491
122						5.376963	4	272	0.0003509	0.0732786
125						9.502991	4	272	0.0000003	0.1226145
126						5.441014	4	272	0.0003147	0.0740869
127						5.510866	4	272	0.0002795	0.0749667
128						12.383544	4	272	0	0.1540557
